# Perfluorocarbon-based artificial oxygen carriers in perioperative and surgical care: a scoping review of basic and translational studies

**DOI:** 10.3389/fmed.2026.1874098

**Published:** 2026-06-24

**Authors:** Xiaojing Guo, Jintao Wei, Bojin Chen, Mao Zhang, Shouyin Jiang, Shanxiang Xu

**Affiliations:** 1 Department of Nursing, The Second Affiliated Hospital of Zhejiang University School of Medicine, Hangzhou, China; 2 Department of Nursing, Zhejiang University School of Medicine, Hangzhou, China; 3 Department of Emergency Medicine, Second Affiliated Hospital, Zhejiang University School of Medicine, Hangzhou, China; 4 Zhejiang Key Laboratory of Trauma, Burn, and Medical Rescue, Hangzhou, China; 5 Zhejiang Province Clinical Research Center for Emergency and Critical Care Medicine, Hangzhou, China; 6 Research Institute of Emergency Medicine, Zhejiang University, Hangzhou, China; 7 National Emergency Medical Rescue Base, Hangzhou, China

**Keywords:** brain injury, hemorrhagic shock, lung injury, organ preservation, oxygen carriers, perfluorocarbon, perioperative care

## Abstract

**Background:**

Perioperative hypoxia remains a common clinical complication. Allogeneic blood transfusion and conventional oxygen support are widely used but limited by blood shortage, supply constraints and ethical challenges. Perfluorocarbon-based oxygen carriers (PFCs) possess superior oxygen solubility and favorable physicochemical features, making promising candidate adjuncts to improve perioperative oxygenation, despite multiple unresolved translational drawbacks.

**Methods:**

This scoping review strictly followed the PRISMA-ScR standard. Six mainstream databases were systematically searched for English human and animal studies published from 2000 to 2025 focusing on perioperative and surgical applications of PFCs; eligible literature was screened and data extracted based on predefined criteria.

**Results:**

A total of 55 publications including 13 randomized controlled trials were enrolled. Preclinical and clinical data demonstrated PFC effectively improves tissue oxygenation and organ protection across hemorrhagic shock, brain injury and acute lung injury, with the most consistent therapeutic gains observed in organ preservation. PFC can reduce allogeneic transfusion requirements during major surgery and benefit cerebral ischemia management, while inflammatory side effects, thrombocytopenia and other adverse reactions have been repeatedly documented.

**Conclusion:**

PFCs show encouraging tissue-protective effects particularly in organ preservation, yet its broad clinical translation is hindered by inherent limitations such as high oxygen dependence, CARPA, reticuloendothelial deposition and historical failure of classic formulations. Further optimized formulation design and large-scale clinical trials are mandatory to improve safety and realize targeted clinical application.

## Introduction

Ensuring oxygen delivery is fundamental to surgical and perioperative care as it underpins cellular metabolism and patient survival (1). Despite advances in perioperative medicine, a range of acute pathological processes such as hemorrhagic shock and acute anemia can critically undermine tissue oxygenation, contributing to organ dysfunction and adverse outcomes (2). These critical conditions create a demand for oxygen delivery during trauma resuscitation, emergency and elective surgery.

The magnitude of this challenge is highlighted by the Global Burden of Disease Study, which reveals that injuries are responsible for 4.3 million deaths annually (3). Despite this substantial demand, most countries are unable to adequately meet their transfusion needs (4, 5). Moreover, the acceleration of global population aging has further shrunk the size of the eligible blood donor population. Conventional blood transfusion practices are further hampered by logistical delays, the risk of transmissible infections, and persistent shortages in blood supply (6).

Several alternative oxygen delivery strategies have been explored to address the limitations of conventional blood transfusion. Hemoglobin-based oxygen carriers (HBOCs) have been extensively investigated as blood substitutes because of their ability to transport oxygen independently of donor blood; however, their clinical development has been hindered by safety concerns, including nitric oxide scavenging, vasoconstriction, and cardiovascular adverse events (7, 8). Extracorporeal extracorporeal membrane oxygenation (ECMO) provide effective cardiopulmonary support through extracorporeal gas exchange and have become established therapies for severe respiratory failure, although their application is limited by invasiveness, technical complexity, and substantial resource requirements (9). Compared with these approaches, perfluorocarbon (PFC)-based oxygen carriers represent a distinct oxygen delivery strategy based on physical oxygen dissolution, offering potential advantages in microvascular oxygen transport, universal compatibility, and long-term storage stability.

In light of these unmet clinical needs, PFCs have attracted substantial research interest due to their unique properties, such as high oxygen solubility, physicochemical stability, and long shelf life (10–13). While preclinical and early clinical studies suggest benefits of PFCs in perioperative and surgical settings, uncertainties remain regarding optimal application, safety, and clinical translatability. To address these gaps, this review systematically examines the evolving evidence from both basic and translational studies, aiming not only to summarize therapeutic efficacy and safety profiles, but also to provide strategic recommendations for advancing the clinical translation of PFCs in perioperative and surgical care.

## Methods

### Search strategy and databases

Guided by the PRISMA-ScR framework, a structured literature search and study selection were performed to comprehensively map the evidence from basic and translational studies (14). We searched the PubMed, Web of Science, Embase, Cochrane Library, Clinical Trials.gov, and Scopus databases for literature published between January 2000 and October 2025, adopting a search strategy combining free-text terms and subject headings. The main search terms were: “(General Surgery OR Surgery OR Surgical Procedures OR Surgical Intervention OR Major Surgery OR Trauma Surgery OR Emergency Surgery OR Critical Surgery OR pre-hospital care OR military medicine) AND (Perfluorocarbon OR PFC OR Fluorocarbon OR Perfluorocarbon-based OR Perfluorocarbon emulsions OR Perfluorodecalin OR Fluosol DA OR Oxygent OR Fluorinated Blood Substitutes OR Perftoran OR Oxyfluor OR Oxycyte)”. Additionally, we manually reviewed the references of relevant original and review articles to identify further pertinent studies. The detailed search strategy and PRISMA-ScR checklist are provided in S1.

### Study selection and screening workflow

Two independent reviewers conducted initial title/abstract screening and full-text evaluation according to predefined inclusion and exclusion criteria. Any disagreements were resolved by consensus discussion or consultation with the corresponding author to reach a final decision.

Inclusion criteria: (1) English-language original articles; (2) Preclinical animal studies, clinical randomized trials, observational studies and case reports focusing on perioperative and surgical application of perfluorocarbon-based oxygen carriers; (3) Published between 2000 and 2025.

Exclusion criteria: (1) Reviews, letters, editorials, conference abstracts and unavailable full-text articles; (2) Studies focusing only on imaging, diabetes or other irrelevant fields; (3) Non-medical application research of perfluorocarbon materials.

### Data extraction and evidence stratification

Author, Nation, Year, Experimental purpose/Indications, Experimental subject, Experimental groups and quantities, PFC product name, and Outcome Assessment were independently extracted by two reviewers. To avoid mixing heterogeneous evidence, we stratified all included studies by study type: preclinical animal studies, randomized controlled trials, observational clinical studies and case reports, and presented them separately in the results section.

Consistent with the standard methodological requirements of PRISMA-ScR scoping review, formal PICOS framework definition, risk-of-bias assessment, study quality scoring, inter-rater agreement statistical analysis and evidence level grading are not mandatory and were not performed in this work.

## Results

**Figure 1:**
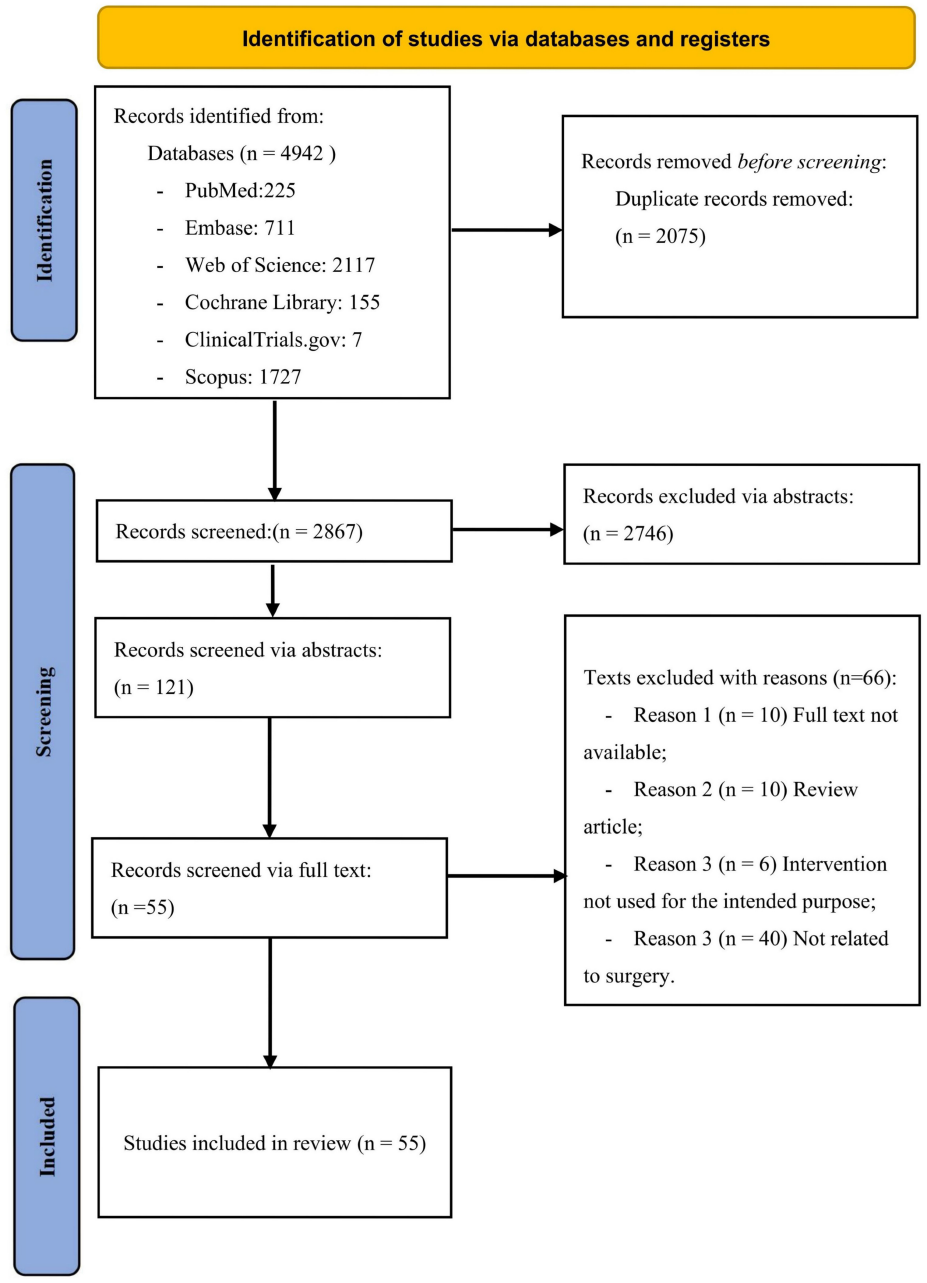
PRISMA review flow chart.

A total of 4,942 records were identified from six databases and registers, including PubMed (*n* = 225). After removal of 2,075 duplicates, 2,867 records entered title and abstract screening. Of these, 2,746 records were excluded because they did not meet the predefined eligibility criteria. The remaining 121 records were assessed in further detail, and 55 studies were finally included (Figure 1). These comprised 32 preclinical animal studies and 23 human studies, including 13 randomized trials. The characteristics of each study are summarized in Table 1. The peaks in publication numbers occurred in 2002, 2006, and 2020. Most studies (46 out of 55) reported positive outcomes. Research has addressed hemorrhagic shock, lung injury, and organ preservation. Table 2 presents the features of the PFCs and their primary applications. Video 1 illustrates the oxygen transport mechanism of PFCs with a frame-by-frame animation.

**Table 1:** Information table of literatures on the application of PFCs in the surgical field.

Reference	Nation	Year	Indications	Subjects	Grouping and sample size	Types of PFCs	Outcome Assessment
Wei Meng et al. (28)	China	2024	Brain injury	ICR mice, SD rats, Rabbits	Total: 50 - Control: Sham (*n* = 10); Model (*n* = 10); PFTBA-L (*n* = 10); GB (*n* = 10). - Intervention: PFTBA-L@GB (*n* = 10).	PFTBA-L@GB nanoparticles	effective
Zheng Peng et al. (31)	China	2024	Brain injury	SAH patients, Male C57BL/6 mice	Total *n* = 224: - Control groups: *n* = 71 - Intervention group: SAH + Saline group: *n* = 36; SAH + PFOC group: *n* = 34.; SAH + O_2_ group: *n* = 34; SAH + PFOC + O_2_ group: *n* = 33; SAH + ML228 group (HIF-1α agonist): *n* = 16.	Perfluorocarbon-based oxygen carrier (PFOC)	effective
Heather F. Pidcoke et al. (66)	USA	2022	Inflammatory response	Baboon model of systemic inflammation	Total: *n* = 25 - Control groups: SAL-SAL: *n* = 4; LPS-SAL: *n* = 4. - Intervention groups: SAL-PFC3: *n* = 5; SAL-PFC12: *n* = 5; LPS-PFC3: *n* = 5; LPS-PFC12: *n* = 2.	Oxycyte	aggravation
Johannes Jägers et al. (46)	Germany	2022	Organ preservation efficacy	kidney perfusion of rats	Total 24: - Control group: *n* = 6. - Intervention groups: 2% A-AOCs group: *n* = 6; 4% A-AOCs group: *n* = 6; 8% A-AOCs group: *n* = 6.	A-AOCs	effective
Maria T. Voelker et al. (37)	Germany	2021	Lung injury	A 20-year-old male refugee with refractory ARDS and influenza A/H1N1 infection	Case report	Perflubron (LiquiVent™, OriGen Biomedical, TX, USA)	Partially effective
Guoyi Wu et al. (47)	China	2021	Organ preservation efficacy	DCD liver graft rats	Total *n* = 18: - Control groups: HTK 3h group *n* = 6; HTK 48h group *n* = 6. - Intervention group *n* = 6.	Oxygenated perfluorocarbon emulsion PFCplus	effective
Jiepei Zhu et al. (13)	USA	2021	Safety and effectiveness verification	healthy juvenile female dorper sheep	Total *n* = 38: - Intervention groups: Oxygent group: *n* = 12; Perftoran group: *n* = 9. - Control groups: Hespan group: *n* = 9; Naive group: *n* = 8.	Oxygent™, Perftoran™(Vidaphor)	effective
Francoise Arnaud et al. (27)	USA	2020	Brain injury	swine fluid percussion injury (FPI) model	Total *n* = 22: - Control groups: SHAM-NON: *n* = 4; TBI-NON: *n* = 6. - Intervention groups: SHAM-NVX: *n* = 4; TBI-NVX: *n* = 8.	Perfluorocarbon emulsion NVX-428 (NuvOx Pharma, Tucson, AZ, USA)	effective
Fusheng Wei et al. (33)	China	2020	Lung injury	dogs	Total: 36 dogs - Control group (CMV): *n* = 12 - Intervention group 1 (NPLV): *n* = 12 - Intervention group 2 (HPLV): *n* = 12	Perfluorochemical (PFC)	effective
Anna Wrobeln et al. (57)	Germany	2020	Normovolemic hemodilution	Male Wistar rats	Total: 16 Control: 8 Intervention: 8	A-AOCs	effective
Sergey Ivanovich Vorobyev et al. (15)	Russia	2020	Safety and effectiveness verification	Patients with severe comorbidities (e.g., acute intestinal bleeding), Dogs, Rabbits	Animal experiments: - Perftoran vs. Polyglucin (dog hemodilutio, Isolated rabbit heart): No explicit *n*; Clinical study (Ftoremulsion III): - Intervention: 32 patients; - Control: Patients using PolyglucinNo explicit *n*.	Perftoran	effective
William C. Culpet al. (32)	USA	2019	Brain injury	adult patients	Total: 24 patients - Control group: *n* = 6 - Intervention group: *n* = 18	DDFPe,NuvOx Pharma, LLC, Tucson, Arizona, USA	effective
Renata Salatti Ferrari et al. (49)	Brazil	2019	Organ preservation efficacy	lung graft cold ischemia rats	Total *n* = 48 rats (6 rats per subgroup): - Control groups: 4 subgroups (LPD 3h, LPD 6h, LPD 12h, LPD 24h). - Intervention groups: 4 subgroups (PFC+LPD 3h, PFC+LPD 6h, PFC+LPD 12h, PFC+LPD 24h).	Vaporized perfluorocarbon (vaporized PFC)	effective
Jia Zhuang et al. (21)	USA	2018	Hemorrhagic shock	male CD-1 mice	Total: 36–48 mice + cell samples (*n* = 3–6/group). - Intervention: RBC–PFC (*n* = 6 for hemorrhagic shock; *n* = 3–6 for safety/biodistribution). - Control: RL (*n* = 6, negative); RBC Vesicle (*n* = 6, cell membrane); PFC Emulsion (*n* = 6, PFC); - Whole Blood (*n* = 6, positive); Vehicle (*n* = 3, safety).	RBC-PFC (biomimetic nanoemulsion)	effective
Alicia M. Bonanno et al. (25)	USA	2018	Hemorrhagic shock	healthy male yorkshire swine	Total *n* = 45: - Control group: *n* = 5. - Intervention groups: *n* = 40 (10 for Hextend, 10 for FFP, 10 for FFP+DDFPe, 10 for FWB).	DDFPe, perfluorocarbon oxygen therapeutic	ineffective
Shreya Bali (38)	UK	2017	Lung injury	A 3-year-old female patient received perfluorocarbon instillation + biodegradable airway stent + bronchoscopy + physiotherapy	Case report	Perfluorocarbon (PFC)	effective
Hui Ding et al. (36)	China	2017	Lung injury	adult patients	Total: 23 patients - Control group: *n* = 11 - Intervention group: *n* = 12	Perfluorohexane (C6F14)	effective
Shinya Okumura et al. (48)	Japan	2017	Organ preservation efficacy	donation after cardiac death (DCD)liver graft rats	Total *n* = 30: Experiment I (30-min warm ischemia): - Control group: (*n* = 5). - Intervention group: (*n* = 5). Experiment II (50-min warm ischemia, survival analysis): - Control group: (*n* = 7). - Intervention group: (*n* = 7).	Oxygent™ (New Alliance, PFC Development, Orlando, FL, USA)	effective
Ashraful Haque et al. (34)	USA	2016	Lung injury	Yorkshire swine	Total *n* = 18: - Intervention groups: PFC post-OA group *n* = 6; PFC pre-OA group: *n* = 6. - Control group: *n* = 6.	Oxycyte (Oxygen Biotherapeutics, Inc., Morrisville, NC, USA)	effective
Mark E. Caridi-Scheible et al. (39)	USA	2016	Lung injury	a 62-year-old man with severe pulmonary hemorrhage post-open pulmonary embolectomy	Case report	Perfluorodecalin	Partially effective
Rania Abutarboush et al. (14)	USA	2016	Safety and effectiveness verification	male sprague-dawley rats	Total *n* = 38: - Intervention group: Perftoran group (*n* = 13). - Control groups: 0.9% NaCl group (crystalloid, *n* = 13); Hextend group (colloid, *n* = 12).	Perftoran	effective
Huan Zhang et al. (29)	China	2015	Brain injury	Sprague-Dawley (SD) rats	Total: 120 rats - Sham operation group (Sham): *n* = 40 - Control group (SAH): *n* = 40 - Intervention group (SAH + PFOB): *n* = 40	Perfluorooctyl-bromide (PFOB)	effective
P.S. Reynolds et al. (26)	USA	2015	Hemorrhagic shock	male new zealand white rabbits	Total *n* = 23: - Control group: Fresh Whole Blood (FWB, autologous), *n* = 5 - Intervention groups: Hextend (HEX) group: *n* = 9; HEX+Perfluorocarbon (PFC) group: *n* = 9.	Oxygent™	ineffective
Christopher M. Horvat et al. (40)	USA	2015	Lung injury	A 17-year-old previously healthy female with severe pulmonary hemorrhage and airway thrombus requiring ECLS	Case report	Perfluorodecalin (PFD)	effective
George Mychaliska et al. (41)	USA	2015	Lung injury	neonates	Total: 16 neonates - Intervention group (PILG): *n* = 8 - Control group (CMV): *n* = 8	Perflubron	Partially effective
Luciana N. Torres et al. (65)	USA	2014	Other diseases: arterial gas embolism (AGE)	male sprague-dawley rats	Total: 14 rats - Control group: *n* = 5 - Oxycyte (C10F_2_0) Group: *n* = 5 - PHER-O_2_ (C10F18) Group: *n* = 4	Oxycyte (C10F_2_0), PHER-O_2_ (C10F18)	effective
T. Marada, K et al. (45)	Czech Republic	2012	Organ preservation efficacy	Rat Pancreas Transplant Model	Total *n* = 24: - Control group: *n* = 8. - Intervention groups: UW group: *n* = 8; PFH group: *n* = 8.	Perfluorohexyloctane (PFH)	effective
Pedro Cabrales et al. (62)	USA	2011	Anemia	hamster window chamber model	Total *n* = 11 (no control group due to lethality of conventional expanders at Hct = 6%): - Subgroup 1 (microvascular measurements): *n* = 6 - Subgroup 2 (cardiac output measurements): *n* = 5	Oxycyte (Synthetic Blood International, Inc., Costa Mesa, CA, USA)	effective
Heide Brandhorst et al. (55)	Sweden	2010	Organ preservation efficacy	Human islets	Total: 23: - Control group (PFD group): *n* = 12. - Intervention group (F6H8S5 group): *n* = 11.	F6H8S5 (perfluorohexyloctane-siloxane 5), perfluorodecalin (PFD)	effective
Aditya Agrawal et al. (44)	UK	2010	Organ preservation efficacy	Porcine Pancreas	Total *n* = 12: - Control group (UW alone):(*n* = 6) - Intervention group (TLM):(*n* = 6)	Perfluorodecalin (manufactured by F2 Chemicals Ltd, Preston, UK)	effective
Naoyuki Hatayama et al. (50)	Japan	2009	Organ preservation efficacy	Rat Heart	Total *n* = 25: - Intervention groups: KH/PFC + CO_2_ *n* = 5; KH/PFC + CO_2_ *n* = 5. - Control groups: UW/PFC + CO_2_ *n* = 5; KH/PFC + CO_2_ *n* = 5; No preservation *n* = 5.	perfluorocarbon	effective
Andrei V. Alexandrov et al. (30)	USA	2008	Brain injury	Adult patients with acute ischemic stroke	Total: 15 patients - Intervention group: *n* = 12 - Control group: *n* = 3	Perflutren-Lipid Microspheres	effective
O.N. Reznik, et al. (53)	Russia	2008	Organ preservation efficacy	kidney grafts	Total: 117 kidney grafts from 61 donors - Donor groups: - Control group (HTK group): *n* = 31. - Intervention group (Perftoran group): *n* = 30. - Recipient groups: - Control group: *n* = 59. - Intervention group: *n* = 58.	Perftoran	effective
Rolf Lindemann et al. (42)	USA	2007	Lung injury	A 2-month-old male infant	Case report	Perfluorodecalin (Flutec P55, F2 Chemicals, UK)	effective
Claes E. G. Lundgren et al. (63)	USA	2006	Anemia	Male Wistar rats	Total 4 experimental series: - Series I (acute fatal anemia): Control (*n* = 8) vs. Intervention (*n* = 8). - Series II (long-term survival): Control (*n* = 8) vs. Intervention (*n* = 8). - Series III (muscle oxygenation in intact rats): Control (*n* = 4) vs. Intervention (*n* = 4). - Series IV (*in vitro* oxygen exchange): Control (*n* = 3) vs. Intervention (*n* = 3)	DDFP nano-emulsion	effective
T. Sakai et al. (51)	Japan	2006	Organ preservation efficacy	Lewis rat islets	Total: *n* = 12 - Control group: *n* = 6 - Intervention group: *n* = 6	Perfluorochemical	effective
D. G. Maluf et al. (52)	USA	2006	Organ preservation efficacy	ACI rats were used as kidney donors	Total m = 24: - Control group (UW group): *n* = 12. - Intervention group (PFC-UW group): *n* = 12.	Perfluorodecalin	effective
Raul C. Verdin-Vasquez et al. (61)	Mexico	2006	Normovolemic hemodilution	Patients undergoing elective cardiac valvuloplasty with CPB	Total *n* = 30: - Intervention group (PFC group): *n* = 15. - Control group: *n* = 15.	Perftoran (commercial name: Perftec, approved in Mexico)	effective
Valérie Jouan-Hureaux et al. (17)	France	2006	Safety and effectiveness verification	peripheral venous blood of healthy volunteers	Total *n* = 3–6/group: - Control: Physiologic: Hct = 40% human blood (*n* = 6). - Dilution: Blood diluted to Hct = 13%/20%/30% with various agents (*n* = 3–6/Hct/agent). - Intervention: 18 comb. of 3 expanders + 2 PFC conc. (4/8 g/dl) at Hct = 13%/20%/30% (*n* = 3–6/comb).	New PFC emulsion (AtoFina, Paris, France)	effective
Naoto Yamamoto et al. (22)	Japan	2005	Hemorrhagic shock	male sprague-dawley rats	Total *n* = 34 (*n* = 7/group for survival; *n* = 5-10/group for other indicators): - Controls: Group I (HS no lavage), Group IV (sham) - Interventions: Group II (HS+N_2_-PFC), Group III (HS+O_2_-PFC)	Perfluorochemical (PFC)	effective
Gregor I. Kemming et al. (24)	Germany	2005	Hemorrhagic shock	beagle dogs	Total *n* = 20: - Control group (COLL): *n* = 10. - Intervention group (PFC): *n* = 10.	Oxygent™	effective
Steven E. Hill et al. (60)	USA	2005	Normovolemic hemodilution	adult patients	Total: 36 patients - Control group: *n* = 11 - Intervention group 1 (Low dose): *n* = 13 - Intervention group 2 (High dose): *n* = 12	AF0144 (Perflubron emulsion, Alliance Pharmaceutical Corp, San Diego, CA, USA)	Partially effective
D. Brandhorst et al. (43)	Germany	2005	Organ preservation efficacy	Pancreases from retired breeder pigs; Islet transplantation model in diabetic nude mice	Total *n* = 24: - Control group:(*n* = 6) - Intervention group: OLM group:(*n* = 8); TLM group:(*n* = 10)	Oxygenated perfluorocarbon (PFC)	effective
Milena Angelova et al. (67)	Japan	2004	Inflammatory response	Sprague-Dawley rats	Total: 36 rats - Control group (CV): *n* = 9 - Intervention group 1 (PLV): *n* = 9 - Intervention group 2 (LPS): *n* = 9 - Intervention group 3 (PLV+LPS): *n* = 9	Perflubron	aggravation
S. Matsumoto et al. (56)	USA	2004	Organ preservation efficacy	Human clinical-grade pancreata	Total *n* = 9: - Control group (UW group): *n* = 6 - Intervention group (TLM group): *n* = 3	Perfluorochemical	effective
D. K. Papadimitriou et al. (64)	Greece	2004	Other diseases: acute intestinal ischemia	New Zealand rabbits	Total: 36 rabbits - Control group: *n* = 12 - Intervention group (PFC-O_2_ group): *n* = 12 - Control subgroup (PFC group): *n* = 12 - Each group was further divided into 3 subgroups by ligated vessels: superior mesenteric artery, mesenteric vein, or both vessels (*n* = 4 per subgroup)	Perfluorodecalin (F-Decalin\C10F18), manufactured by Fluoron GmbH, Neu-Ulm	effective
Markus Paxian et al. (23)	USA	2003	Hemorrhagic shock	male sprague-dawley rats	Total *n* = 56: - Control groups: Sham (A): *n* = 8; HAES (B): *n* = 8; WB (C): *n* = 8; PRBCs (D): *n* = 8. - Intervention groups: PFC-2.7 (E): *n* = 8; PFC-5.4 (F): *n* = 8; PRBCs+PFC (G): *n* = 8.	Oxygent™	effective
RONALD B. HIRSCHL et al. (35)	USA	2002	Lung injury	adult patients	Total: 90 patients - Intervention group (PLV): *n* = 65 - Control group (CMV): *n* = 25	Perflubron (LiquiVent)	Partially effective
Steven E. Hill et al. (58)	USA	2002	Normovolemic hemodilution	Adult patients undergoing elective CABG surgery with CPB	Total: *n* = 36 - Control group (electrolyte solution): *n* = 11; - Intervention groups: AF0144 low-dose (1.8 g PFC/kg): *n* = 13; - AF0144 high-dose (2.7 g PFC/kg): *n* = 12.	AF0144 (Perflubron emulsion, Alliance Pharmaceutical Corp, San Diego, CA, USA)	effective
Donat R. Spahn et al. (59)	Switzerland	2002	Normovolemic hemodilution	adult patients	Total *n* = 492: - Intervention group (PFC group): *n* = 241. - Control group (standard of care): *n* = 251. - Subgroup (target population): *n* = 330.	Perflubron emulsion (Oxygent™, Alliance Pharmaceutical Corp., San Diego, CA, USA)	effective
Robert J. Frumento et al. (19)	USA	2002	Safety and effectiveness verification	adult patients	Total: 9 patients - Intervention group (PFC): *n* = 4 - Control group: *n* = 5	Perflubron emulsion (Oxygent™)	effective
Jun Sakanoue et al. (18)	Japan	2001	Safety and effectiveness verification	male wistar rats	Total *n* = 21: - Intervention group: Neo-PFC group (*n* = 7). - - Control groups: Fluosol-DA group (*n* = 7); BSA-Buffer group (*n* = 7).	newly developed perfluorocarbon emulsion, Fluosol-DA	effective
Dirk Nolte et al. (16)	USA	2000	Safety and effectiveness verification	Male Syrian golden hamsters	Total: 36 - Intervention subgroups (ANH + colloid + perflubron emulsion): *n* = 6 each - Control subgroups (ANH + colloid alone): *n* = 6 each - Additional hypervolemic infusion groups: *n* = 6 each	Perflubron emulsion (Oxygent, AF0144)	effective
SHINICHI et al. (54)	Japan	2000	Organ preservation efficacy	Human patients undergoing whole-pancreas transplantation	Total *n* = 54 - Control group (UW alone group): *n* = 44 - Intervention group (Two-layer method group): *n* = 10	Perfluorochemical	effective
Phillip T. Leese et al. (20)	USA	2000	Safety and effectiveness verification	healthy human volunteers	Total *n* = 48: - Control group: *n* = 16. - Intervention groups: P1.2 group (*n* = 16); P1.8 group (*n* = 16).	Oxygent™	effective

**Table 2:** Characteristics of primary PFCs included in the review.

Generation classification	PFC product	Particle size	Core component	Half–life (Approx.)	Emulsification method	Primary scenarios
First generation	Fluosol–DA	< 0.2 μm	Perfluorodecalin + Perfluorotripropylamine	−24 h (Human)	Mechanical Emulsification (Pluronic F−68 + Egg Yolk Phospholipid)	Coronary angioplasty, intraoperative transfusion, temporary oxygen supply for acute hypoxia, suppression of lung injury inflammation, treatment of carbon monoxide poisoning
First generation	Perftoran (Vidaphor)	0.1–0.2 μm	Perfluorodecalin + Perfluoromethylcyclohexylpiperidine	−24 h (Human)	High–Pressure Homogenization (Proxanol 268)	Hemorrhagic shock resuscitation, organ preservation, COVID−19–related hypoxia, hemodilution in cardiac valve replacement surgery, treatment of maxillofacial space infections, kidney transplant perfusion
Second generation	Oxygent™ (AF0144)	0.16–0.18 μm	Perflubron (Perfluorooctyl bromide)	−6–9 h (Human, RES Clearance)	High–Pressure Homogenization (Egg Yolk Phospholipid)	Surgical hemodilution, CPB, allogeneic blood transfusion alternative in non–cardiac surgery, hemorrhagic shock resuscitation, gastrointestinal ischemia protection, oxygen supply support in coronary artery bypass grafting
Second generation	Oxycyte	−0.2 μm	tert–Butylperfluorocyclohexane	Unknown	High-Pressure Homogenization (Egg Yolk Phospholipid)	Acute ischemic stroke, TBI, hemodilution, protection against ischemia–reperfusion injury, treatment of arterial gas embolism
Second generation	PFH (Perfluorohexyloctane)	N/A	Perfluorohexyloctane	Unknown	Mechanical Emulsification (Lecithin)	Pancreatic preservation, improvement of islet transplant survival, alleviation of ischemia–reperfusion injury during pancreatic cold storage
Second generation	F6H8S5	N/A	Perfluorohexyloctane–siloxane 5	Unknown	Mechanical Emulsification (Egg Yolk Phospholipid)	Human pancreatic preservation, enhancement of islet oxygenation, improvement of post–transplant function, increase of islet ATP content
Second generation	Perflubron	0.16–0.18 μm	Perflubron (Perfluorooctyl bromide)	−6–9 h (Human)	High–Pressure Homogenization (Egg Yolk Phospholipid)	Treatment of ARDS, liquid ventilation for lung injury, treatment of postoperative atelectasis, promotion of lung development in neonates with congenital diaphragmatic hernia
Third generation	DDFPe	< 300 nm (Nano–scale)	DDFP	−90 min (Human)	Nanoemulsification (Human Serum Albumin Stabilization)	Acute ischemic stroke, TBI, hemorrhagic shock resuscitation, emergency treatment of anemia, tissue preconditioning protection
Third generation	A–AOCs (Albumin–derived)	< 220 nm	Albumin + Perfluorodecalin	Unknown	Ultrasonic Emulsification	Organ preservation (kidney/liver/lung), massive hemodilution, oxygen supply for ischemic tissues, perfusion protection of transplanted organs
Third generation	RBC–PFC	−170 nm	RBC Membrane + PFC	Unknown	Ultrasonic Emulsification (3 min)	Hemorrhagic shock resuscitation, maintenance of vascular stability, protection of liver and spleen tissues, oxygen supply without immune activation
Third generation	PFTBA–L@GB	Nano–scale	Perfluorotributylamine + Ginkgolide B	Unknown	Self–Assembly	Ischemic stroke (synergistic intervention of thrombosis and inflammation), reduction of cerebral infarction volume, inhibition of platelet aggregation, neuroprotection
Third generation	PFOB (Perfluorooctyl bromide)	Nano–scale	Perfluorooctyl bromide	3–4 days (Human)	High–Pressure Homogenization (Egg Yolk Phospholipid)	Protection against early brain injury after subarachnoid hemorrhage, alleviation of ischemia–reperfusion injury, protection of hepatocyte mitochondrial function
Third generation	Perfluorodecalin (PFD)	N/A	Perfluorodecalin	7–8 days (Tissue)	Mechanical Emulsification (Lecithin)	Prolongation of intestinal tissue viability in acute intestinal ischemia, clearance of airway thrombi in pulmonary hemorrhage, assisted ventilation for neonatal respiratory distress syndrome

**Figure 2:**
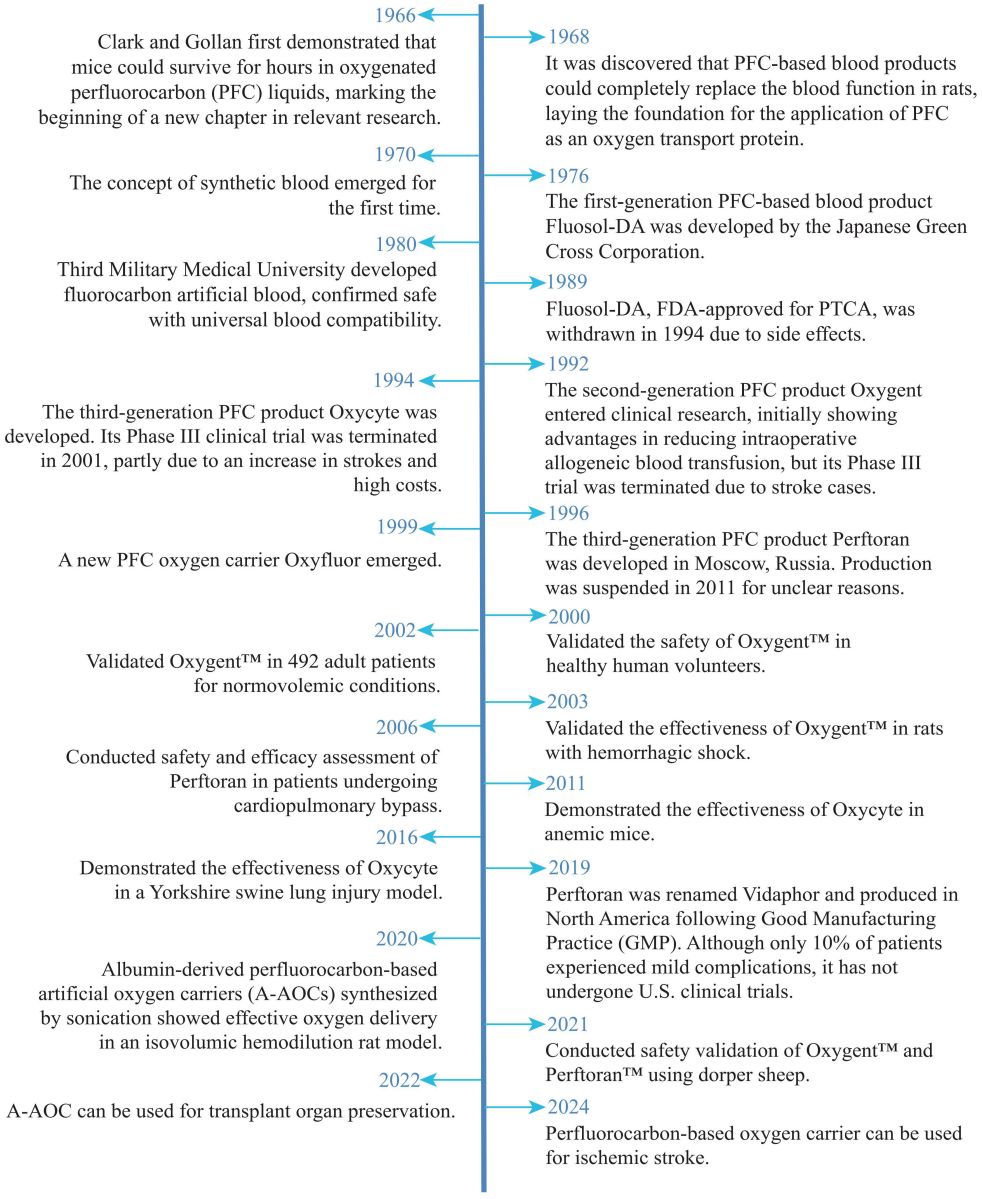
Event timeline of PFC-based oxygen carriers.

The search was limited to studies published from 2000 onward because the objective of this scoping review was to map contemporary preclinical and translational evidence relevant to perioperative and surgical applications of PFC-based oxygen carriers. However, given their historical importance, key pre-2000 milestones and early PFC product development are summarized separately in Supplemental File 2. The development of PFCs has followed key milestones, which are summarized in Figure 2.

### Physicochemical performance and translational safety of PFCs

Across nine studies, the safety and functional performance of PFCs have been thoroughly validated. Evaluation begins with animal experiments, where the oxygen-carrying efficiency and gas transport properties of PFCs were confirmed. In animal models, administration of high-dose oxygent or perftoran led only to transient and reversible thrombocytopenia, without evidence of platelet activation or leukocyte recruitment. Shortening the infusion duration further mitigated early adverse effects, demonstrating good hematologic tolerance (15). The rat study showed that repeated Perftoran exposure resulted in minimal dural artery constriction, slight arteriolar diameter changes, and mild transient increases in blood pressure without causing anemia, indicating cerebrovascular safety (16). Furthermore, PFCs provided superior preservation of myocardial and tissue oxygenation compared to standard plasma substitutes in severe hypoxia (17). In hamster models, intravital fluorescence microscopy demonstrated that perfluorobromide emulsions are compatible with hydroxyethyl starch, gelatin, and human serum albumin in the microcirculation (18).

*In vitro* hemorheological assessments revealed that PFCs formulated with albumin, hydroxyethyl starch, or modified gelatin maintained near-physiological plasma and whole-blood viscosity at low hematocrit, without adversely affecting erythrocyte deformability (19). Oxygen kinetics studies further showed that novel PFCs can preserve mitochondrial cytochrome oxidase function at reduced oxygen fractions and achieve greater arterial oxygen solubility compared to Fluosol-DA, underscoring its improved oxygen delivery capabilities (20).

Clinical research further substantiates these findings. During cardiac surgery, intravenous administration of perfluorobromide emulsion helped to preserve gastric intramucosal pH and CO_2_ levels and shortened postoperative bowel transit time, suggesting protective effects on splanchnic perfusion and gastrointestinal motility (21). Randomized controlled trials in healthy volunteers demonstrated that perfluorobrominated alkanes did not impair immune function. Only mild and transient influenza-like symptoms were observed at higher doses, supporting a favorable safety and tolerability profile (22).

### Therapeutic efficacy of PFCs in hemorrhagic shock resuscitation models

In animal models of hemorrhagic shock, PFCs demonstrate variable therapeutic effects. In a rat study, red blood cell membrane-coated PFCs biomimetic nanocarriers maintained mean arterial pressure at approximately 75 mmHg. Their efficacy was comparable to autologous whole blood and superior to Ringer's solution (23). PFCs peritoneal lavage increased 48-h survival rates to 85.7% compared to 14.3% in controls among male Sprague-Dawley (SD) rats with severe hemorrhagic shock. Treatment also corrected metabolic acidosis, preserved intestinal blood flow, and minimized mucosal injury (24). In a 1-h hemorrhagic shock model in SD rats, perfluorooctyl bromide emulsion combined with hydroxyethyl starch preserved hepatic sinusoidal perfusion and elevated hepatic oxygen tension, providing greater liver protection than either hydroxyethyl starch or stored red blood cells alone (25). In beagle dogs, Oxygent™ at a dosage of 4.5 mL/kg combined with 6% hydroxyethyl starch and hyperoxia increased dissolved oxygen content and reduced oxygen debt, surpassing the effects observed with hydroxyethyl starch alone (26).

However, certain formulations have not demonstrated benefit in larger animal models. In Yorkshire pigs with lactate levels near 7.0 mmol/L, dodecafluoropentane (DDFP) emulsion combined with fresh frozen plasma (FFP) failed to improve 180-min survival. Several animals died soon after administration, likely due to rapid pulmonary intravascular macrophage responses triggered by the nanoparticles (27). Similarly, in New Zealand white rabbits, the combination of hextend and PFCs delayed peak mean arterial pressure and increased methemoglobin levels. There was no improvement in hemodynamics, probably due to coagulation disturbances and inflammatory dysregulation induced by the combined formulation (28). The described results indicate promising efficacy for certain PFCs in small animal models, while highlighting significant challenges related to safety and efficacy in larger species.

### Neuroprotective potential of PFCs in acute brain injury and ischemic events

Animal studies indicate that PFCs and nanoparticle-based formulations can substantially attenuate both structural and functional brain injuries by promoting local oxygen delivery. In a porcine traumatic brain injury model, low-dose PFCs reduced cerebellar spongiform changes, decreased the number of ischemic neurons, and lowered the presence of Fluoro-Jade B-positive Purkinje cells (29). Only a transient elevation in pulmonary artery pressure was observed. In addition, in ischemic stroke animal models, nanoparticles loaded with ginkgolide B inhibited platelet adhesion by 85.49% and suppressed aggregation by 8.3%. These nanoparticles also significantly reduced cerebral infarction volume from 57.90% to 5.03% (30). In a subarachnoid hemorrhage model, Perfluorooctyl bromide (PFOB) nanoparticles lowered brain water content from 80.65% to 78.40% and alleviated neuronal injury in the hippocampal Cornu Ammonis 1 region (31).

Clinical studies further support the feasibility and therapeutic potential of PFCs in cerebral ischemia. In a cohort of 15 acute ischemic stroke patients, ultrasound-activated perfluorobutane microbubbles administered with tPA increased middle cerebral artery recanalization rates to 50%, compared to 18% with tPA alone. Importantly, no symptomatic intracranial hemorrhage was reported (32). PFCs also improved neurological outcomes in patients with subarachnoid hemorrhage as well as in related animal models. Biomarker analysis in cerebrospinal fluid revealed an AUC of 0.8254 for poor prognosis prediction (33). Moreover, a phase Ib/II trial demonstrated that high-dose DDFPe improved functional outcomes at 30 and 90 days in acute ischemic stroke. No maximum tolerated dose was determined, and adverse events were similar to those in the placebo group (34).

### Pulmonary applications and the role of PFCs in hypoxic lung injury

Animal studies provide consistent evidence that PFCs improve oxygenation, lung mechanics, and inflammatory markers in models of acute lung injury, with efficacy closely linked to the timing of administration. In a canine model of acute lung injury, partial liquid ventilation with PFCs resulted in enhanced oxygenation and increased lung compliance compared with conventional mechanical ventilation. This intervention also elevated the IL-10 to TNF-α ratio, suggesting a reduction in pulmonary inflammation (35). In Yorkshire pigs with oleic acid-induced lung injury, intravenous Oxygent administered after injury increased arterial oxygen content, reduced pulmonary edema, and achieved a median survival of 240 min. Conversely, administration prior to injury shortened survival to 87.5 min, highlighting the detrimental effects associated with inappropriate timing (36).

Clinical investigations suggest that the therapeutic effects of PFCs in lung injury are heterogeneous and depend on the patient population as well as the underlying cause. In a randomized trial involving 90 adults with ARDS or acute lung injury, Perflubron partial liquid ventilation did not significantly affect mortality or ventilator-free days compared to conventional ventilation, although a subgroup of patients younger than 55 years in the perflubron group experienced more rapid weaning (37). Some beneficial outcomes have been reported in specific clinical contexts. In 23 patients with burns and smoke inhalation, intratracheal perfluorohexane improved lung function and lowered IL-6 and TNF-α levels (38). Isolated case studies have shown that perflubron perfusion under extracorporeal membrane oxygenation improved refractory H1N1-related ARDS ventilation (39). Furthermore, PFCs lavage combined with a biodegradable stent successfully resolved atelectasis in a three-year-old child (40).

Nevertheless, not all experiences have been favorable. perfluorodecalin (PFD) lavage failed to control severe postoperative pulmonary hemorrhage in a 62-year-old patient (41). In contrast, perfluorinated naphthalene lavage effectively removed distal airway thrombi and supported lung re-expansion in a 17-year-old patient on extracorporeal life support (ECLS) (42). Pediatric applications have produced mixed results. While perflubron-induced lung growth enhanced pulmonary development in neonates with congenital diaphragmatic hernia, these patients had lower survival rates compared to those receiving conventional ventilation (43). In another instance, PFD lavage improved pulmonary function without adverse effects in a child with Niemann-Pick C_2_ disease (44).

### Organ preservation advancements with PFCs

Animal studies provide strong evidence that PFCs enhance the viability and function of multiple organs by improving oxygen delivery and maintaining metabolic stability. For pancreatic preservation, use of a PFC-based one-layer method resulted in a higher islet equivalent yield after 7 h in retired breeding pig pancreata than the traditional two-layer method (45). However, mechanistic analysis showed no difference in intrapancreatic PFCs concentration between the two-layer method and the University of Wisconsin (UW) solution group after 24 h in porcine pancreata. This suggests the observed benefit may arise from enhanced carbon dioxide absorption rather than improved oxygen delivery (46). Further work in Brown Norway rat pancreas transplantation showed that pancreata preserved for 18 h with perfluorohexyloctane had lower TNF-α and endothelin-1 mRNA expression relative to the UW solution group. This was accompanied by reduced pancreatic enzyme release and less ischemia-reperfusion injury (47).

Renal models have also demonstrated significant benefits. In an *ex vivo* normothermic rat perfusion model, a 4% albumin-derived PFCs maintained higher glomerular filtration rates of 300 ± 36 μl/min/g, compared to 156 ± 37 μl/min/g in controls, and reduced tissue injury (48). In studies of liver preservation, PFCs maintained ATP levels and provided greater protection against liver injury compared with histidine-tryptophan-ketoglutarate (HTK) solution in SD rat donation after cardiac death (DCD) transplantation (49). Preoxygenated UW solution raised fourteen-day survival rates to 85.7%, compared to 28.6% in controls (50). In Wistar rats, combining vaporized PFCs with low-potassium dextran (LPD) solution during lung preservation increased superoxide dismutase activity at 3 to 6 h, while reducing interstitial inflammation and atelectasis (51). For heart preservation, immersion of isolated rat hearts in PFCs with KH perfusion and carbon dioxide modification enabled successful preservation up to 96 h, with 100% recovery at 72 h and 75% at 96 h, outperforming UW-based methods (52). In islet transplantation, oxygenated PFCs improved success rates in Lewis rats, with five out of six recipients achieving engraftment compared to one out of six in controls (53). Oxygenated PFCs also activated cytoprotective responses during 24-h kidney preservation (54).

Clinical studies confirm the translational value of PFCs preservation, especially in improving graft function and yield. In a cohort of 117 DCD kidney transplant pairs, *in situ* perfusion with oxygenated perftoran increased immediate graft function to 64.41% from 32.76% in controls, while reducing delayed graft function from 67.24% to 35.59%. There was also a twofold greater reduction in serum creatinine by postoperative day twenty-one (55). In pancreas transplantation, among 54 whole-organ transplants, the two-layer method preserved ten edema-free grafts, whereas the UW group preserved none, and 90% of recipients achieved insulin independence during hospitalization (56). Comparative human pancreas studies demonstrated that F6H8S5 provided superior intrapancreatic oxygen tension, ATP content, and islet yield compared to PFD (57). In a nine clinical-grade pancreas preservation study, the two-layer method produced a yield of 3,415 ± 227 IE/g after a mean storage time of 11.7 ± 2.0 h, significantly higher than the UW method, which achieved 2,006 ± 337 IE/g after 4.8 ± 0.5 h. This resulted in three successful islet transplantations (58).

### The normovolemic hemodilution strategy with PFCs

Animal studies have established that PFCs can effectively reduce the need for allogeneic blood transfusions during normovolemic hemodilution by supporting oxygen delivery in diluted blood. In a male Wistar rat model of massive hemodilution, albumin-derived perfluorocarbon-based artificial oxygen carriers (A-AOCs) prolonged survival, maintained mean arterial pressure and pH, lowered blood lactate levels, and reduced ischemic injury in the small intestine, as indicated by decreased Chiu scores. Furthermore, A-AOCs downregulated renal EPO-mRNA, a hypoxia marker, thereby providing protection against hypoxic tissue damage (59).

Clinical studies further corroborate the benefits of PFCs-supported normovolemic hemodilution. In a phase II trial of 36 patients undergoing coronary artery bypass grafting, AF0144 in combination with normovolemic hemodilution at a hematocrit of 20–22% increased cerebral blood flow. Only 33% of patients in the high-dose group met transfusion triggers, compared to 91% in the control group (60). In a multicenter study of 492 patients undergoing non-cardiac surgery with significant blood loss, PFCs-based hemodilution reduced the median transfusion volume to 2 units versus 4 units in controls, and resulted in transfusion avoidance for 26% of patients, compared to 16% in the control group (61). In 36 cardiac surgery patients under cardiopulmonary bypass, low-dose AF0144 increased cerebral blood flow without embolic risk, while the high-dose group experienced a greater incidence of embolism, indicating a dose-dependent effect (62). A randomized trial of 30 patients undergoing cardiac valve repair also found that those receiving Perftoran had significantly higher intraoperative arterial oxygen partial pressure, reduced intraoperative demand for allogeneic red blood cell transfusion, and no complications or mortality (63).

### Therapeutic roles of PFCs in severe anemia and other diseases

Animal studies consistently demonstrate that PFCs significantly enhance tissue oxygenation and protect against ischemic injury in severe anemia and other acute disease states. These effects are mediated by the high oxygen transport capacity of PFCs. In hamster models of profound anemia with hematocrit reduced to 6%, administration of Oxygent combined with 100% inspired oxygen maintained mean arterial pressure and cardiac output. This intervention preserved systemic oxygen delivery and oxygen consumption in conditions that are typically fatal when treated with conventional plasma expanders (64). In lethally anemic rats, infusion of a 2% DDFP nanoemulsion increased skeletal muscle oxygen tension by 50% to 100% for about two and a half hours at hemoglobin concentrations of only 1.3 to 1.6 g/dl. As a result, all treated rats survived, while control animals with hemoglobin around 2.8 g/dl experienced complete mortality (65). Evidence of PFCs' efficacy extends beyond anemia to other ischemic conditions. In a rabbit model of acute intestinal ischemia involving 8 h of mesenteric vessel ligation, oxygenated F-Decalin produced only mild mucosal injury, in stark contrast to the extensive necrosis observed in controls and in non-oxygenated PFCs groups. This protective effect was associated with significantly smaller increases in serum lactate dehydrogenase and creatine phosphokinase levels (66). In models of severe arterial gas embolism, administration of PFCs restored small artery blood flow to 80% to 85% of baseline. In comparison, saline-treated animals exhibited only about 11% of baseline flow. PFCs treatment also accelerated gas bubble absorption (67).

### Immunological considerations with inflammatory sequelae

Animal studies consistently show that adverse effects associated with PFCs administration may be significantly amplified in the presence of systemic inflammation. In a primate model of lipopolysaccharide-induced systemic inflammation, treatment with PFCs induced delayed and prolonged thrombocytopenia. This change was accompanied by reduced clot strength and elevated microsomal content. Animals developed diffuse microvascular hemorrhage, which led to death. These outcomes were linked to the activation of the mononuclear phagocyte system and increased platelet consumption driven by inflammation (68). Further evidence from an endotoxemic rat model indicates that PFCs did not suppress pulmonary inflammation. On the contrary, it led to additional upregulation of IL-6 transcription. These findings suggest that, when PFCs are administered in an inflammatory environment, cytokine responses may be exacerbated rather than attenuated (69).

## Discussion

This review synthesizes preclinical and clinical research demonstrating that PFCs exert favorable tissue-protective effects under hypoxic perioperative conditions, with the most robust translational gains observed in organ preservation settings, while adverse inflammatory risks rise under systemic inflammatory priming.

PFCs possess a strongly electronegative hydrocarbon backbone, granting them chemical inertness and an oxygen solubility approximately 20-fold greater than water (10–12, 70). Due to their inherent hydrophobicity, they require emulsification for intravenous administration (71, 72). Unlike hemoglobin's chemical binding, PFCs transport oxygen primarily via physical dissolution according to Henry's law, strictly relying on high inspired oxygen fractions (FiO_2_) for optimal efficacy (73, 74). Benefiting from their ultra-fine particle size, PFC emulsions can penetrate ischemic microcirculation inaccessible to erythrocytes, and circulating PFCs are mainly eliminated via pulmonary exhalation following reticuloendothelial system (RES) phagocytosis (75, 76). Despite these unique properties, clinical translation has been constrained by historical failures. Classic formulations, including Fluosol-DA and Oxygent, were discontinued due to unsatisfactory safety profiles and a lack of definitive clinical superiority (70, 73). Current translational hurdles involve dose-dependent thrombocytopenia, complement activation-related pseudoallergy (CARPA), pulmonary adverse events, and prolonged hepatic-splenic RES accumulation, which can exaggerate inflammatory responses under endotoxemia. Consequently, promising preclinical data cannot overestimate clinical prospects.

In real-world settings, PFCs may reduce the need for allogeneic transfusions in acute anemia and major surgery by supporting oxygen delivery at low hematocrit levels. Furthermore, PFCs can serve as a temporary alternative for oxygen delivery when blood products are unavailable, particularly in prehospital emergency settings. Unlike normal saline, which lacks oxygen-carrying capacity and can cause acidosis or edema during large-volume resuscitation, PFCs offer urgent oxygen support. Nevertheless, hemodilution limits their capacity for sustained oxygen delivery, and the optimal dosing regimens remain uncertain.

Notably, inflammation exacerbation after PFCs therapy may potentially be associated with activation of Toll-like receptors (TLRs), particularly TLR2 and TLR4, on monocytes/macrophages. In immune-primed or endotoxemic conditions, PFCs particle and their metabolites might act as danger-associated molecular patterns, possibly triggering MyD88-dependent NF-κB cascades and subsequent pro-inflammatory cytokine release. It should be noted that relevant mechanistic evidence remains partially speculative, and some available evidence is derived from environmental PFAS toxicological studies rather than clinically applied medical-grade PFC emulsions (75, 76). TLR-mediated signaling may act synergistically with CARPA after complement opsonization, amplifying inflammatory responses as PFC droplets accumulate in the liver and lungs. Prolonged hepatic retention of larger PFC particles also aggravates oxidative stress and metabolic disturbance, potentially contributing to liver dysfunction under inflammatory conditions (74, 75, 77). In clinical settings, CARPA and mild inflammatory fluctuations are the main documented adverse reactions of approved PFC products, whereas definitive TLR-mediated inflammatory injury induced by medical PFC emulsions still lacks solid clinical and *in vivo* verification. Further well-designed basic studies are required to clarify whether clinical PFCs can directly trigger TLR-dependent inflammatory pathways under physiological and inflammatory conditions.

The most consistent translational benefits identified in this review were observed in organ preservation. As modern transplantation increasingly relies on machine technologies including hypothermic oxygenated perfusion (HOPE) and normothermic machine perfusion (NMP), PFCs should be viewed as oxygen-carrying adjuncts that enhance oxygen availability within preservation solutions, rather than as competing technologies. The future clinical role of PFCs likely lies in complementing existing oxygen therapeutic platforms for targeted tissue oxygenation (9, 78).

Despite valuable insights, several limitations remain. Most existing studies focus on short-term outcomes, with limited data on long-term human efficacy and no unified clinical standards for PFC use in organ preservation. Furthermore, human trials are predominantly small-sample pilot studies. The absence of reliable dose-oxygen delivery predictive models, coupled with inherent limitations like high oxygen dependence and adverse reactions, hinders the development of clinical guidelines.

Future research on PFCs should prioritize four areas. First, formulation optimization must enhance biomimetic nanocarrier stability and reduce immunogenicity; innovations like RBC membrane-stabilized nanocarriers (23) or DDFP microbubbles (65) show promise. Second, personalized medicine approaches should identify biomarkers (e.g., inflammatory cytokines or TLR expression) to stratify patients and guide therapy (68, 75). Third, combination strategies such as pairing PFCs with anti-platelet agents (30), anti-inflammatory drugs, or colloids should be further explored. Fourth, large-scale multicenter trials are essential to confirm long-term effectiveness and safety, focusing on potential cumulative toxicity after repeated administration. Additionally, developing lyophilized PFCs could improve shelf-life, enhance portability, and reduce oxygen requirements during transfusion, expanding their potential for military and prehospital applications (79, 80). From a regulatory perspective, standardized manufacturing processes and predefined safety endpoints are essential before broader clinical implementation.

## Conclusion

This scoping review summarizes available preclinical and clinical evidence confirming the favorable tissue-protective effects of PFC across multiple perioperative hypoxic conditions including hemorrhagic shock, brain injury and acute lung damage, with the most robust therapeutic outcomes observed in various organ preservation scenarios. PFC formulations can effectively reduce perioperative allogeneic transfusion consumption under low hematocrit conditions. Nevertheless, multiple inherent drawbacks and historical developmental obstacles substantially limit widespread clinical translation. As documented in previous clinical trials, discontinued classic products such as Fluosol-DA and Oxygent failed routine clinical promotion mainly due to dose-dependent thrombocytopenia, CARPA, pulmonary adverse events, persistent hepatic and splenic reticuloendothelial deposition and systemic inflammatory exacerbation under inflammatory status, alongside the mandatory requirement for high-concentration inspired oxygen to exert efficacy. Current available evidence mostly derives short-term experimental data and small-sized single-center clinical trials without unified administration specifications. Future research should focus on formulation optimization, biomarker-based individualized medication, combined therapeutic regimens and large multicenter clinical verification, and the development of lyophilized PFC preparations to expand pre-hospital and military application potential. PFCs represent an adjunct for managing perioperative hypoxia, though rigorous translational efforts are essential to realize their full clinical potential.

## Data Availability

The original contributions presented in the study are included in the article/Supplementary material, further inquiries can be directed to the corresponding authors.

## Supplementary material

Supplementary Video 1 Oxygen-carrying mechanism of PFCs.
