# Supplementary material for: Perfluorocarbon-based artificial oxygen carriers in perioperative and surgical care: a scoping review of basic and translational studies
Source: Front Med (Lausanne). 2026 Jun 24;13:1874098. doi: 10.3389/fmed.2026.1874098 (PMC13343355; doi:10.3389/fmed.2026.1874098)
Supplement: Supplementary file 1 [file Supplementary_File_1.docx]

**Search Strategy**

**PubMed**

| Search  number | Search Details | Filters | Results |
| --- | --- | --- | --- |
| 5 | 1 AND 2 | Case Reports, Clinical Trial, Clinical Trial, Phase I, Clinical Trial, Phase II, Clinical Trial, Phase III, Clinical Trial, Phase IV, Observational Study, Randomized Controlled Trial, Clinical Trial, Veterinary, from 2000 - 2025 | 225 |
| 4 | 1 AND 2 | from 2000 - 2025 | 989 |
| 3 | 1 AND 2 |  | 1,356 |
| 2 | (((((((((General Surgery[MeSH Terms]) OR (Surgery[Title/Abstract])) OR (Surgical Procedures[Title/Abstract])) OR (Surgical Intervention[Title/Abstract])) OR (Major Surgery[Title/Abstract])) OR (Trauma Surgery[Title/Abstract])) OR (Emergency Surgery[Title/Abstract])) OR (Critical Surgery[Title/Abstract])) OR (pre-hospital care[Title/Abstract])) OR (military medicine[Title/Abstract]) |  | 1,801,816 |
| 1 | (((((((((((Perfluorocarbon[MeSH Terms]) OR (PFC[Title/Abstract])) OR (Fluorocarbon[Title/Abstract])) OR (Perfluorocarbon-based[Title/Abstract])) OR (Perfluorocarbon emulsions[Title/Abstract])) OR (Perfluorodecalin[Title/Abstract])) OR (Fluosol DA[Title/Abstract])) OR (Oxygent[Title/Abstract])) OR (Fluorinated Blood Substitutes[Title/Abstract])) OR (Perftoran[Title/Abstract])) OR (Oxyfluor[Title/Abstract])) OR (Oxycyte[Title/Abstract]) |  | 36,644 |

**Cochrane Library**

ID Search Hits

#1 MeSH descriptor: [Fluorocarbons] explode all trees 238

#2 (PFC):ti,ab,kw OR (Fluorocarbon):ti,ab,kw OR (Perfluorocarbon-based):ti,ab,kw OR (Perfluorocarbon emulsions):ti,ab,kw OR (Perfluorodecalin):ti,ab,kw 1843

#3 (Fluosol DA):ti,ab,kw OR (Oxygent):ti,ab,kw OR (Fluorinated Blood Substitutes):ti,ab,kw OR (Perftoran):ti,ab,kw OR (Oxyfluor):ti,ab,kw 24

#4 (Oxycyte):ti,ab,kw 1

#5 #1 OR #2 OR #3 #4 2047

#6 MeSH descriptor: [General Surgery] explode all trees 519

#7 (Surgery):ti,ab,kw OR (Surgical Procedures):ti,ab,kw OR (Surgical Intervention):ti,ab,kw OR (Major Surgery):ti,ab,kw OR (Trauma Surgery):ti,ab,kw 333672

#8 (Emergency Surgery):ti,ab,kw OR (Critical Surgery):ti,ab,kw OR (pre-hospital care):ti,ab,kw OR (military medicine):ti,ab,kw 11806

#9 #6 OR #7 OR #8 334629

#10 #5 AND #9 191

2000-2025

English 155
