# Supplementary material for: Perfluorocarbon-based artificial oxygen carriers in perioperative and surgical care: a scoping review of basic and translational studies
Source: Front Med (Lausanne). 2026 Jun 24;13:1874098. doi: 10.3389/fmed.2026.1874098 (PMC13343355; doi:10.3389/fmed.2026.1874098)
Supplement: Supplementary file 2 [file Supplementary_File_2.docx]

**Supplemental File 2**

**Development history**

The evolution of PFC-based blood products provides critical context for understanding the heterogeneity observed in the included studies. Early-generation products such as Fluosol-DA and Oxygent demonstrated proof-of-concept for oxygen transport yet were limited by side effects and formulation instability (1-4). Subsequent advancements, including Oxycyte, Perftoran, and albumin-stabilized formulations, have achieved improved biocompatibility and oxygen-dissolving performance (5, 6). These historical developments help explain why recent studies, particularly in organ preservation and controlled ischemia models, demonstrate more consistent and favorable outcomes than earlier trials.

In 1966, Clark and Gollan first demonstrated that mice could survive for several hours in oxygenated PFC liquids, thus opening a new chapter in research (7). In 1968, the discovery that PFC -based blood products could completely substitute for rat blood functions laid the foundation for the application of PFC as an oxygen transporter (8) . In 1970, the concept of synthetic blood emerged for the first time (2) .

In 1976, the first-generation PFC-based blood product Fluosol - DA was developed by Japan's Green Cross Corporation (3). In 1980, Chinese scientists from the Shanghai Institute of Organic Chemistry and Third Military Medical University pioneered fluorocarbon-based artificial blood, successfully transfusing two patients without adverse reactions, confirming its universal blood type compatibility. Fluosol-DA gained FDA approval for coronary angioplasty in 1989 but was withdrawn in 1994 due to side effects (9). In 1992, the second-generation PFC product Oxygent emerged and entered the clinical research phase. Initially, it demonstrated the advantage of reducing the need for donated blood transfusions during surgery. However, due to the occurrence of stroke cases, the phase III clinical trial of this product was eventually terminated. (4).

In 1999, the novel PFC-based oxygen carrier Oxyfluor emerged (10). The third-generation PFC include Oxycyte (Oxygen Biotherapeutics Inc., Morrisville, NC) and Perftoran (Scientific Production Company Perftoran, Russia) . In 1994, Oxycyte was developed by a company in San Diego, California. Its phase III trial was terminated in 2001, partly due to increased strokes and high costs. In 2002, Oxygent™ was validated in 492 adult patients under normovolemic conditions for its safety(11).

Perftoran was developed in Moscow, Russia in 1996 and approved for clinical use in Russia and Mexico from 2005 to 2010. It has been administered to over 35,000 patients, demonstrating evidence of significant benefits alongside relatively minor and manageable adverse reactions . In 2006, the safety and efficacy of Perftoran were assessed in patients undergoing cardiopulmonary bypass(12). However, in 2011, Russia suspended its production for unclear reasons(13). In 2016, Oxycyte’s effectiveness was demonstrated in a Yorkshire swine lung injury model(14). In 2019, Perftoran was renamed Vidaphor and produced and sold in North America in compliance with the Good Manufacturing Practice of Medical Products . Only 10% of patients experienced mild complications, yet no clinical trials have been conducted in the United States (4). A 2020 breakthrough introduced an albumin-stabilized PFC oxygen carrier , demonstrating effective oxygen delivery in a rat model (15). These advancements have propelled the development of PFC-based blood substitutes. Additionally, in 2020, an albumin-derived PFC-based artificial oxygen carrier (A-AOCs) synthesized by sonication showed effective oxygen delivery in an isovolemic hemodilution rat model(6).

In 2021, the safety of Oxygent™ and Perftoran™ was validated using Dorper sheep(5). In 2022, A-AOCs was shown to be applicable for transplant organ preservation(16). Looking ahead to 2024, PFC-based oxygen carriers are being explored for use in ischemic stroke(17). These advancements have propelled the development of PFC-based blood substitutes.

**Chemical structure and physical properties**

The physicochemical features of PFC directly shape the patterns observed in our results—especially the superiority in organ preservation and selective effectiveness in hemorrhagic shock and neuroprotection.

PFC are synthetic hydrocarbon derivatives with the general formula CₙF₂ₙ₊₂, typically containing 8 to 12 carbon atoms. Structurally, they feature a carbon chain framework surrounded by fluorine atoms, whose high electronegativity confers exceptional chemical stability. This inertness ensures PFC remain unaltered in vivo, resisting metabolism or decomposition, thus exhibiting robust chemical inertness and metabolic stability (9, 18) . Due to this characteristic, PFC emulsifiers serve as excellent solvents for gases, particularly demonstrating remarkable solubility for oxygen and carbon dioxide (9, 18, 19) .

PFC typically exist as colorless, odorless, and transparent liquids at room temperature (18) , and its density is approximately twice that of water (18, 20), and its surface tension is very low (15~18 dynes/cm at 25°C) (18). This property enhances their capacity to dissolve and release gases in liquid media. PFC exhibit extraordinary gas-dissolving capabilities: at 37°C and standard atmospheric pressure, their oxygen solubility reaches 20-fold that of water and 2~3-fold that of blood(18) . However, due to the poor water miscibility of PFC, emulsification is generally required for its application (9, 21) .

The boiling point of PFC is determined by carbon chain length: shorter-chain PFC (e.g., C₄F₁₀) exhibit lower boiling points (-2°C), whereas longer-chain analogs (e.g., C₅F₁₂) have higher boiling points (29°C) (19) . This makes them easy to lyophilize and thermally sterilize (22) .

**Oxygen-binding capacity and biocompatibility**

The distinction between physical oxygen dissolution in PFC and chemical binding in hemoglobin is crucial for interpreting the results. For example, studies achieving high oxygenation environments consistently reported improved outcomes, whereas models with inadequate oxygen supplementation demonstrated diminished efficacy. This discrepancy parallels clinical concerns that PFC performance is highly dependent on FiO₂ (9, 23), explaining both the strong results in ischemia models and the weaker or inconsistent results in inflammatory and pulmonary injury models.

The oxygen transport mechanism of PFC differs significantly from that of hemoglobin. Hemoglobin forms stable complexes with oxygen through chemical binding, whereas PFC carry oxygen via physical dissolution (24) . Oxygen dissolution in PFC adheres to Henry's law, stipulating that at a constant temperature, the equilibrium concentration of dissolved oxygen is directly proportional to the partial pressure of the gas. This principle endows PFC with the ability to rapidly and extensively release oxygen in response to physiological demands (9, 25) . Moreover, with dimensions approximately 100-fold smaller than red blood cells, PFC molecules can effectively perfuse ischemic regions that are otherwise inaccessible to erythrocyte-rich blood (9, 23) . This unique characteristic allows PFC to significantly elevate blood oxygen content under hypoxic conditions, ensuring adequate tissue oxygenation.

PFC exhibit favorable biocompatibility in vivo. The carbon-fluorine bonds in PFC molecules are highly stable, preventing them from readily reacting with other substances. This inertness ensures that PFC do not easily participate in chemical reactions within biological environments, thereby reducing potential toxicity and side reactions (26) . PFC can be naturally eliminated through the respiratory system after residing in the body for a period, reducing risks caused by long-term accumulation (19, 25) .

**The half-life of perfluorinated carbon blood products**

The short intravascular half-life observed in most PFC emulsions provides a mechanistic explanation for why PFC is effective as perioperative temporizing agents, for example, as bridges for anemia (27, 28), intraoperative hemodilution (6, 11), or acute neuroprotection (29, 30). Conversely, prolonged retention of long-chain fluorocarbons informs the observed adverse reactions in systemic inflammation models (31).

Unlike hemoglobin that binds oxygen, PFC-based oxygen carriers depend on physical dissolution, thus requiring high inspired oxygen fractions (70-100%) to ensure sufficient oxygen transport (24) .Thus, clinical use of PFC-based artificial blood necessitates simultaneous inhalation of high-concentration oxygen to fully and promptly exert its function. Such application conditions impose operational limitations, increasing rescue preparation time in emergency scenarios.

PFC blood products exhibit remarkable differences in half-life due to their distinct components and ratios. The blood half-life of most PFC emulsions is less than 24 hours, and the terminal half-life of DDFPE in humans is only about 90 minutes (19), Fluosol has a blood half-life of 1-2 days, perfluorooctyl bromide has an elimination half-life of 3~4 days, and perfluorodichlorooctane shows an 8-day elimination half-life (32) . The blood half-life of the Fluosol DA-20 formulation is 24 hours (33) . Conversely, perfluorotripropylamine, a PFC with a long half-life, remains in the body for up to 65 days (33).Blood products composed of PFC in different proportions balance stability and metabolic rate through component modulation, but such combinations further exacerbate therapeutic risks.Take Fluosol-DA as an example. It is a PFC blood product mixed with perfluorotripropylamine and perfluorodecalin (with a tissue half-life of 7-8 days). The stability is increased by perfluorotripropylamine, but it cannot completely eliminate the hidden danger of perfluorotripropylamine remaining in the body for a long time, and there is also the possibility of adverse reactions caused by accumulation. Therefore, when preparing PFC blood products, the half-life of different fluorides should be taken into account to achieve the optimal proportion.

**Emulsion conditions**

Variability in droplet size, surfactant composition, emulsification method, and dosing were major contributors to the heterogeneity of study outcomes. Studies using optimized nanoemulsions often demonstrated superior oxygen delivery and tissue protection (6, 29) , consistent with the principle that small, stable emulsions (< 200 nm) reduce RES clearance and improve vascular persistence.

An ideal blood substitute should have a high oxygen-carrying capacity, maintain long-term stability, be free of adverse reactions, be able to reach all areas of the human body, be rapidly metabolized and eliminated, and maintain stable blood pressure. Although PFC do not affect human metabolism, they need to be emulsified into tiny particles (<0.2 μm) to exist more stably in the blood (34) . Studies (35) have shown that the optimal results of optimizing emulsion preparation parameters are as follows: the emulsification time is set to 8-10 min, the emulsification pressure is controlled at 5000 psi, the PFC concentration is determined to be 10% v/v (under this concentration, the particle size of the emulsion is < 220 nm, enabling sterile filtration), and the surfactant concentration is 2% w/v, specifically adopting a combination of 1% Pluronic F-68 and 1% Pluronic F-127 (this concentration ensures the smallest particle size of the emulsion and controllable toxicity).

As the first-generation PFC blood product, Fluosol-DA-20 is generally well-tolerated by patients when the infusion volume is less than 500 ml, without severe side effects (33) . This dosage selection, which takes into account the patient's body weight, disease severity, and the rapidity of infusion, aims to ensure oxygen supply while minimizing the occurrence of side effects. The multiple administration dosage of Fluosol DA-20 can reach 56 ml/kg (36). The effective dosage of Oxygent is 1.35 g/kg. While some studies have indicated that its infusion may increase the incidence of stroke, there are also studies confirming the relative stability of its physiological parameters (9) .

PFC emulsions are typically administered via intravenous infusion, offering the advantages of rapidity and effectiveness, especially in emergency and trauma care settings. It is recommended that the infusion be performed in a high-concentration oxygen environment to facilitate oxygen dissolution in PFC and achieve optimal oxygen-carrying effects (37) .

1. Lc C, F G. Survival of mammals breathing organic liquids equilibrated with oxygen at atmospheric pressure. Science (New York, NY). 1966;152(3730).

2. Clark LC, Jr., Kaplan S, Becattini F, Benzing G, 3rd. Perfusion of whole animals with perfluorinated liquid emulsions using the Clark bubble-defoam heart-lung machine. Federation proceedings. 1970;29(5):1764-70.

3. Lowe KC. Fluosol®: The First Commercial Injectable Perfluorocarbon Oxygen Carrier. . Academic Press. 2006.

4. Jahr JS, Guinn NR, Lowery DR, Shore-Lesserson L, Shander A. Blood Substitutes and Oxygen Therapeutics: A Review. Anesth Analg. 2021;132(1):119-29.

5. Zhu J, Parsons JT, Yang Y, Martin E, Brophy DF, Spiess BD. Platelet and White Cell Reactivity to Top-Load Intravenous Perfluorocarbon Infusion in Healthy Sheep. J Surg Res. 2021;267:342-9.

6. Wrobeln A, Jagers J, Quinting T, Schreiber T, Kirsch M, Fandrey J, et al. Albumin-derived perfluorocarbon-based artificial oxygen carriers can avoid hypoxic tissue damage in massive hemodilution. Scientific reports. 2020;10(1):11950.

7. Clark LC, Jr., Gollan F. Survival of mammals breathing organic liquids equilibrated with oxygen at atmospheric pressure. Science. 1966;152(3730):1755-6.

8. Geyer RP, Monroe, R.C., and Taylor, K. in Organ Perfusion and Preservation. Appleton, Century and Crofts, New York. 1968:pp. 85 - 97.

9. Zhao M, Liu H, Jahr JS. Perfluorocarbon-based oxygen carriers: What is new in 2024? Journal of Anesthesia and Translational Medicine. 2024;3(1):10-3.

10. Briceño JC, Rincón IE, Vélez JF, Castro I, Arcos MI, Velásquez CE. Oxygen transport and consumption during experimental cardiopulmonary bypass using oxyfluor. ASAIO journal (American Society for Artificial Internal Organs : 1992). 1999;45(4):322-7.

11. Spahn DR, Waschke KF, Standl T, Motsch J, Van Huynegem L, Welte M, et al. Use of perflubron emulsion to decrease allogeneic blood transfusion in high-blood-loss non-cardiac surgery - Results of a European phase 3 study. Anesthesiology. 2002;97(6):1338-49.

12. Verdin-Vasquez RC, Zepeda-Perez C, Ferra-Ferrer R, Chavez-Negrete A, Contreras F, Barroso-Aranda J. Use of perftoran emulsion to decrease allogeneic blood transfusion in cardiac surgery: clinical trial. Artificial cells, blood substitutes, and immobilization biotechnology. 2006;34(4):433-54.

13. Latson GW. Perftoran (Vidaphor)-Introduction to Western Medicine. Shock. 2019;52(1S Suppl 1):65-9.

14. Haque A, Scultetus AH, Arnaud F, Dickson LJ, Chun S, McNamee G, et al. The Emulsified PFC Oxycyte((R)) Improved Oxygen Content and Lung Injury Score in a Swine Model of Oleic Acid Lung Injury (OALI). Lung. 2016;194(6):945-57.

15. Wrobeln A, Jägers J, Quinting T, Schreiber T, Kirsch M, Fandrey J, et al. Albumin-derived perfluorocarbon-based artificial oxygen carriers can avoid hypoxic tissue damage in massive hemodilution. Scientific Reports. 2020;10(1):11950.

16. Jagers J, Kirsch M, Cantore M, Karaman O, Ferenz KB. Artificial oxygen carriers in organ preservation: Dose dependency in a rat model of ex-vivo normothermic kidney perfusion. Artif Organs. 2022;46(9):1783-93.

17. Peng Z, Ye QS, Li XJ, Zheng DY, Zhou Y, Hang CH, et al. Novel perfluorocarbon-based oxygenation therapy alleviates Post-SAH hypoxic brain injury by inhibiting HIF-1alpha. Free Radic Biol Med. 2024;214:173-83.

18. Nocentini G, MacLaren G, Bartlett R, De Luca D, Perdichizzi S, Stoppa F, et al. Perfluorocarbons in Research and Clinical Practice: A Narrative Review. ASAIO Journal. 2023;69(12):1039-48.

19. Johnson JLH, Unger E. Dodecafluoropentane emulsion as an oxygen therapeutic. Artificial Cells, Nanomedicine, and Biotechnology. 2024;52(1):462-75.

20. Nb C, F C, Mm T, P P, Dk C, A C, et al. A new era in oxygen therapeutics? From perfluorocarbon systems to haemoglobin-based oxygen carriers. Blood reviews. 2022;54.

21. Jaegers J, Haferkamp S, Arnolds O, Moog D, Wrobeln A, Nocke F, et al. Deciphering the Emulsification Process to Create an Albumin-Perfluorocarbon-(o/w) Nanoemulsion with High Shelf Life and Bioresistivity. Langmuir. 2022;38(34):10351-61.

22. Bialas C, Moser C, Sims CA. Artificial oxygen carriers and red blood cell substitutes: A historic overview and recent developments toward military and clinical relevance. J Trauma Acute Care Surg. 2019;87(1S Suppl 1):S48-s58.

23. Haldar R, Gupta D, Chitranshi S, Singh MK, Sachan S. Artificial Blood: A Futuristic Dimension of Modern Day Transfusion Sciences. Cardiovascular & Hematological Agents in Medicinal Chemistry. 2019;17(1):11-6.

24. Jahr JS, Walker V, Manoochehri K. Blood substitutes as pharmacotherapies in clinical practice. Current Opinion in Anaesthesiology. 2007;20(4):325-30.

25. Lambert E, Janjic JM. Quality by design approach identifies critical parameters driving oxygen delivery performance in vitro for perfluorocarbon based artificial oxygen carriers. Scientific Reports. 2021;11(1):5569.

26. Jägers J, Wrobeln A, Ferenz KB. Perfluorocarbon-based oxygen carriers: from physics to physiology. Pflugers Archiv: European Journal of Physiology. 2021;473(2):139-50.

27. Cabrales P, Carlos Briceno J. Delaying Blood Transfusion in Experimental Acute Anemia with a Perfluorocarbon Emulsion. Anesthesiology. 2011;114(4):901-11.

28. Lundgren CE, Bergoe GW, Tyssebotn IM. Intravascular fluorocarbon-stabilized microbubbles protect against fatal anemia in rats. Artificial cells, blood substitutes, and immobilization biotechnology. 2006;34(5):473-86.

29. Meng W, Ye H, Ma Z, Liu L, Zhang T, Han Q, et al. Perfluorocarbon Nanoparticles Incorporating Ginkgolide B: Artificial O(2) Carriers with Antioxidant Activity and Antithrombotic Effect. ChemMedChem. 2024;19(1):e202300312.

30. Arnaud F, Haque A, Morris ME, Moon-Massat P, Auker C, Biswajit S, et al. Treatment of Swine Closed Head Injury with Perfluorocarbon NVX-428. Med Sci (Basel). 2020;8(4).

31. Pidcoke HF, Delacruz W, Herzig MC, Schaffer BS, Leazer ST, Fedyk CG, et al. Perfluorocarbons cause thrombocytopenia, changes in RBC morphology and death in a baboon model of systemic inflammation. PLoS One. 2022;17(12):e0279694.

32. Spahn DR. Blood substitutes. Artificial oxygen carriers: perfluorocarbon emulsions. Critical Care (London, England). 1999;3(5):R93-7.

33. Kaufman RJ. Medical oxygen transport using perfluorochemicals. Biotechnology (Reading, Mass). 1991;19:127-62.

34. Riess JG. Oxygen carriers ("blood substitutes")--raison d'etre, chemistry, and some physiology. Chemical reviews. 2001;101(9):2797-920.

35. Fraker CA, Mendez AJ, Inverardi L, Ricordi C, Stabler CL. Optimization of perfluoro nano-scale emulsions: the importance of particle size for enhanced oxygen transfer in biomedical applications. Colloids Surf B Biointerfaces. 2012;98:26-35.

36. Hall JE, Karlson KH, DuRant RH. Massive transfusion of perfluorocarbon emulsion (Fluosol-DA) in newborn piglets. Developmental Pharmacology and Therapeutics. 1987;10(3):163-73.

37. Hill SE. Perfluorocarbons: Knowledge Gained From Clinical Trials. Shock. 2019;52(1S):60-4.

**Online Supplementary File S2: Development history, physicochemical properties, and translational characteristics of perfluorocarbon-based oxygen carriers**
