# Supplementary material for: Perfluorocarbon-based artificial oxygen carriers in perioperative and surgical care: a scoping review of basic and translational studies
Source: Front Med (Lausanne). 2026 Jun 24;13:1874098. doi: 10.3389/fmed.2026.1874098 (PMC13343355; doi:10.3389/fmed.2026.1874098)
Supplement: Supplementary file 3 [file Table_1.DOCX]

## Authors’ contributions

Study design and conception: all authors

Literature search and selection: CB, LS, JK

Data extraction and synthesis: CB, LS

Writing: CB, JK

Critical revision: CB, PRMR, TL, JK

Approved the final version of the manuscript: all authors
