# Supplementary material for: Perfluorocarbon-based artificial oxygen carriers in perioperative and surgical care: a scoping review of basic and translational studies
Source: Front Med (Lausanne). 2026 Jun 24;13:1874098. doi: 10.3389/fmed.2026.1874098 (PMC13343355; doi:10.3389/fmed.2026.1874098)
Supplement: Supplementary file 4 [file Table_2.DOCX]

**Data availability statement**

The datasets used during this study are available from the corresponding author upon

reasonable request.
