# Supplementary material for: Perfluorocarbon-based artificial oxygen carriers in perioperative and surgical care: a scoping review of basic and translational studies
Source: Front Med (Lausanne). 2026 Jun 24;13:1874098. doi: 10.3389/fmed.2026.1874098 (PMC13343355; doi:10.3389/fmed.2026.1874098)
Supplement: Supplementary file 6 [file Table_4.DOCX]

**Table 1.Information table of literatures on the application of PFCs in the surgical field..**

| **Author** | **Nation** | **Year** | **Indications** | **Subjects** | **Grouping and Sample size** | **Types of PFCs** | **Outcome Assessment** |
| --- | --- | --- | --- | --- | --- | --- | --- |
| Wei Meng et al.(28) | China | 2024 | Brain injury | ICR mice、SD rats、Rabbits | Total: 50 - Control:Sham (n=10);Model (n=10);PFTBA-L (n=10);GB (n=10). - Intervention: PFTBA-L@GB (n=10). | PFTBA-L@GB nanoparticles | effective |
| Zheng Peng et al.(31) | China | 2024 | Brain injury | SAH patients、Male C57BL/6 mice | Total n=224: - Control groups: : n=71 - Intervention group:SAH + Saline group: n=36;SAH + PFOC group: n=34.;SAH + O₂ group: n=34;SAH + PFOC + O₂ group: n=33; SAH + ML228 group (HIF-1α agonist): n=16. | Perfluorocarbon-based oxygen carrier (PFOC) | effective |
| Heather F. Pidcoke et al.(66) | USA | 2022 | Inflammatory response | Baboon model of systemic inflammation | Total: n=25 - Control groups:SAL-SAL: n=4;LPS-SAL: n=4. - Intervention groups:SAL-PFC3 : n=5;SAL-PFC12: n=5;LPS-PFC3: n=5;LPS-PFC12: n=2. | Oxycyte | aggravation |
| Johannes Jägers et al. (46) | Germany | 2022 | Organ preservation efficacy | kidney perfusion of rats | Total 24: - Control group:n=6. - Intervention groups:2% A-AOCs group: n=6;4% A-AOCs group: n=6;8% A-AOCs group: n=6. | A-AOCs | effective |
| Maria T. Voelker et al.(37) | Germany | 2021 | Lung injury | A 20-year-old male refugee with refractory ARDS and influenza A/H1N1 infection | Case report | Perflubron (LiquiVent™, OriGen Biomedical, TX, USA) | Partially effective |
| Guoyi Wu et al.(47) | China | 2021 | Organ preservation efficacy | DCD liver graft rats | Total n=18: - Control groups:HTK 3h group n=6;HTK 48h group n=6. - Intervention group n=6:. | Oxygenated perfluorocarbon emulsion PFCplus | effective |
| Jiepei Zhu et al.(13) | USA | 2021 | Safety and effectiveness verification | healthy juvenile female dorper sheep | Total n=38: - Intervention groups:Oxygent group: n=12;Perftoran group: n=9. - Control groups:Hespan group: n=9; Naive group: n=8. | Oxygent™、Perftoran™（Vidaphor） | effective |
| Francoise Arnaud et al.(27) | USA | 2020 | Brain injury | swine fluid percussion injury (FPI) model | Total n=22: - Control groups:SHAM-NON: n=4;TBI-NON: n=6. - Intervention groups:SHAM-NVX: n=4;TBI-NVX: n=8. | Perfluorocarbon emulsion NVX-428 (NuvOx Pharma, Tucson, AZ, USA) | effective |
| Fusheng Wei et al.(33) | China | 2020 | Lung injury | dogs | Total: 36 dogs - Control group (CMV): n=12 - Intervention group 1 (NPLV): n=12 - Intervention group 2 (HPLV): n=12 | Perfluorochemical (PFC) | effective |
| Anna Wrobeln et al(57) | Germany | 2020 | Normovolemic hemodilution | Male Wistar rats | Total: 16 Control:8 Intervention: 8 | A-AOCs | effective |
| Sergey Ivanovich Vorobyev et al.(15) | Russia | 2020 | Safety and effectiveness verification | Patients with severe comorbidities (e.g., acute intestinal bleeding)、Dogs、Rabbits | Animal experiments: - Perftoran vs. Polyglucin (dog hemodilutio、Isolated rabbit heart): No explicit n; Clinical study (Ftoremulsion III): - Intervention: 32 patients; - Control: Patients using PolyglucinNo explicit n. | Perftoran | effective |
| William C. Culpet al.(32) | USA | 2019 | Brain injury | adult patients | Total: 24 patients - Control group: n=6 - Intervention group: n=18 | DDFPe,NuvOx Pharma, LLC, Tucson, Arizona, USA | effective |
| Renata Salatti Ferrari et al.(49) | Brazil | 2019 | Organ preservation efficacy | lung graft cold ischemia rats | Total n=48 rats (6 rats per subgroup): - Control groups: 4 subgroups (LPD 3h, LPD 6h, LPD 12h, LPD 24h). - Intervention groups: 4 subgroups (PFC+LPD 3h, PFC+LPD 6h, PFC+LPD 12h, PFC+LPD 24h). | Vaporized perfluorocarbon (vaporized PFC) | effective |
| Jia Zhuang et al.(21) | USA | 2018 | Hemorrhagic shock | male CD-1 mice | Total: 36 - 48 mice + cell samples (n = 3 - 6/group). Intervention: RBC - PFC (n = 6 for hemorrhagic shock; n = 3 - 6 for safety/biodistribution). Control:RL (n = 6, negative);RBC Vesicle (n = 6, cell membrane);PFC Emulsion (n = 6, PFC); Whole Blood (n = 6, positive);Vehicle (n = 3, safety). | RBC-PFC (biomimetic nanoemulsion) | effective |
| Alicia M. Bonanno et al.(25) | USA | 2018 | Hemorrhagic shock | healthy male yorkshire swine | Total n=45: - Control group: n=5. - Intervention groups: n=40 (10 for Hextend, 10 for FFP, 10 for FFP+DDFPe, 10 for FWB). | DDFPe, perfluorocarbon oxygen therapeutic | ineffective |
| Shreya Bali(38) | UK | 2017 | Lung injury | A 3-year-old female patient received perfluorocarbon instillation + biodegradable airway stent + bronchoscopy + physiotherapy | Case report | Perfluorocarbon (PFC) | effective |
| Hui Ding et al.(36) | China | 2017 | Lung injury | adult patients | Total: 23 patients - Control group: n=11 - Intervention group: n=12 | Perfluorohexane (C6F14) | effective |
| Shinya Okumura et al.(48) | Japan | 2017 | Organ preservation efficacy | donation after cardiac death (DCD)liver graft rats | Total n=30: Experiment I (30-min warm ischemia): - Control group:(n=5). - Intervention group:(n=5). Experiment II (50-min warm ischemia, survival analysis): - Control group:(n=7). - Intervention group:(n=7). | Oxygent™ (New Alliance, PFC Development, Orlando, FL, USA) | effective |
| Ashraful Haque et al.(34) | USA | 2016 | Lung injury | Yorkshire swine | Total n=18: - Intervention groups:PFC post-OA group n=6;PFC pre-OA group: n=6. - Control group:n=6. | Oxycyte (Oxygen Biotherapeutics, Inc., Morrisville, NC, USA) | effective |
| Mark E. Caridi-Scheible et al.(39) | USA | 2016 | Lung injury | a 62-year-old man with severe pulmonary hemorrhage post-open pulmonary embolectomy | Case report | Perfluorodecalin | Partially effective |
| Rania Abutarboush et al.(14) | USA | 2016 | Safety and effectiveness verification | male sprague-dawley rats | Total n=38: - Intervention group: Perftoran group (n=13). - Control groups: 0.9% NaCl group (crystalloid, n=13);Hextend group (colloid, n=12). | Perftoran | effective |
| Huan Zhang et al.(29) | China | 2015 | Brain injury | Sprague-Dawley (SD) rats | Total: 120 rats - Sham operation group (Sham):n=40 - Control group (SAH):n=40 - Intervention group (SAH + PFOB):n=40 | Perfluorooctyl-bromide (PFOB) | effective |
| P.S. Reynolds et al.(26) | USA | 2015 | Hemorrhagic shock | male new zealand white rabbits | Total n=23: - Control group: Fresh Whole Blood (FWB, autologous), n=5 - Intervention groups:Hextend (HEX) group: n=9;HEX+Perfluorocarbon (PFC) group: n=9. | Oxygent™ | ineffective |
| Christopher M. Horvat et al.(40) | USA | 2015 | Lung injury | A 17-year-old previously healthy female with severe pulmonary hemorrhage and airway thrombus requiring ECLS | Case report | Perfluorodecalin (PFD) | effective |
| George Mychaliska et al.(41) | USA | 2015 | Lung injury | neonates | Total: 16 neonates - Intervention group (PILG): n=8 - Control group (CMV): n=8 | Perflubron | Partially effective |
| Luciana N. Torres et al.(65) | USA | 2014 | Other diseases:arterial gas embolism (AGE) | male sprague-dawley rats | Total: 14 rats - Control group:n=5 - Oxycyte (C₁₀F₂₀) Group:n=5 - PHER-O₂ (C₁₀F₁₈) Group:n=4 | Oxycyte (C₁₀F₂₀)、PHER-O₂ (C₁₀F₁₈) | effective |
| T. Marada, K et al.(45) | Czech  Republic | 2012 | Organ preservation efficacy | Rat Pancreas Transplant Model | Total n=24: - Control group:n=8. - Intervention groups:UW group:n=8;PFH group:n=8. | Perfluorohexyloctane (PFH) | effective |
| Pedro Cabrales et al.(62) | USA | 2011 | Anemia | hamster window chamber model | Total n=11 (no control group due to lethality of conventional expanders at Hct=6%): - Subgroup 1 (microvascular measurements): n=6 - Subgroup 2 (cardiac output measurements): n=5 | Oxycyte (Synthetic Blood International, Inc., Costa Mesa, CA, USA) | effective |
| Heide Brandhorst et al.(55) | Sweden | 2010 | Organ preservation efficacy | Human islets | Total: 23: - Control group (PFD group): n=12. - Intervention group (F6H8S5 group): n=11. | F6H8S5 (perfluorohexyloctane-siloxane 5)、perfluorodecalin (PFD) | effective |
| Aditya Agrawal et al.(44) | UK | 2010 | Organ preservation efficacy | Porcine Pancreas | Total n=12: - Control group (UW alone):(n=6) - Intervention group (TLM):(n=6) | Perfluorodecalin (manufactured by F2 Chemicals Ltd, Preston, UK) | effective |
| Naoyuki Hatayama et al.(50) | Japan | 2009 | Organ preservation efficacy | Rat Heart | Total n=25: - Intervention groups: KH/PFC + CO₂ n=5;KH/PFC + CO₂ n=5. - Control groups:UW/PFC + CO₂ n=5;KH/PFC + CO₂ n=5;No preservation n=5. | perfluorocarbon | effective |
| Andrei V. Alexandrov et al.(30) | USA | 2008 | Brain injury | Adult patients with acute ischemic stroke | Total: 15 patients - Intervention group:n=12 - Control group: n=3 | Perflutren-Lipid Microspheres | effective |
| O.N. Reznik, et al.(53) | Russia | 2008 | Organ preservation efficacy | kidney grafts | Total: 117 kidney grafts from 61 donors 1. Donor groups: - Control group (HTK group):n=31. - Intervention group (Perftoran group):n=30. 2. Recipient groups: - Control group:n=59. - Intervention group:n=58. | Perftoran | effective |
| Rolf Lindemann et al.(42) | USA | 2007 | Lung injury | A 2-month-old male infant | Case report | Perfluorodecalin (Flutec P55, F2 Chemicals, UK) | effective |
| Claes E. G. Lundgren et al.(63) | USA | 2006 | Anemia | Male Wistar rats | Total 4 experimental series: - Series I (acute fatal anemia): Control (n=8) vs. Intervention (n=8). - Series II (long-term survival): Control (n=8) vs. Intervention (n=8). - Series III (muscle oxygenation in intact rats): Control (n=4) vs. Intervention (n=4). - Series IV (in vitro oxygen exchange): Control (n=3) vs. Intervention (n=3) | DDFP nano-emulsion | effective |
| T.Sakai et al.(51) | Japan | 2006 | Organ preservation efficacy | Lewis rat islets | Total: n=12 - Control group: n=6  - Intervention group:n=6 | Perfluorochemical | effective |
| D.G. Maluf et al.(52) | USA | 2006 | Organ preservation efficacy | ACI rats were used as kidney donors | Total m=24: - Control group (UW group):n=12. - Intervention group (PFC-UW group):n=12. | Perfluorodecalin | effective |
| Raul C. Verdin-Vasquez et al.(61) | Mexico | 2006 | Normovolemic hemodilution | Patients undergoing elective cardiac valvuloplasty with CPB | Total n=30: - Intervention group (PFC group): n=15. - Control group: n=15. | Perftoran (commercial name: Perftec, approved in Mexico) | effective |
| Valérie Jouan-Hureaux et al.(17) | France | 2006 | Safety and effectiveness verification | peripheral venous blood of healthy volunteers | Total n = 3 - 6/group: Control:Physiologic: Hct = 40% human blood (n = 6). Dilution: Blood diluted to Hct = 13%/20%/30% with various agents (n = 3 - 6/Hct/agent). Intervention: 18 comb. of 3 expanders + 2 PFC conc. (4/8 g/dL) at Hct = 13%/20%/30% (n = 3 - 6/comb.). | New PFC emulsion (AtoFina, Paris, France) | effective |
| Naoto Yamamoto et al.(22) | Japan | 2005 | Hemorrhagic shock | male sprague-dawley rats | Total n=34 (n=7/group for survival; n=5-10/group for other indicators): - Controls: Group I (HS no lavage)、Group IV (sham) - Interventions: Group II (HS+N₂-PFC)、Group III (HS+O₂-PFC) | Perfluorochemical (PFC) | effective |
| Gregor I. Kemming et al.(24) | Germany | 2005 | Hemorrhagic shock | beagle dogs | Total n=20: - Control group (COLL): n=10. - Intervention group (PFC): n=10. | Oxygent™ | effective |
| Steven E. Hill et al.(60) | USA | 2005 | Normovolemic hemodilution | adult patients | Total: 36 patients - Control group: n=11 - Intervention group 1 (Low dose): n=13 - Intervention group 2 (High dose): n=12 | AF0144 (Perflubron emulsion, Alliance Pharmaceutical Corp, San Diego, CA, USA) | Partially effective |
| D. Brandhorst et al.(43) | Germany | 2005 | Organ preservation efficacy | Pancreases from retired breeder pigs;Islet transplantation model in diabetic nude mice | Total n=24: - Control group:(n=6) - Intervention group:OLM group:(n=8);TLM group:(n=10) | Oxygenated perfluorocarbon (PFC) | effective |
| Milena Angelova et al.(67) | Japan | 2004 | Inflammatory response | Sprague-Dawley rats | Total: 36 rats - Control group (CV): n=9 - Intervention group 1 (PLV): n=9 - Intervention group 2 (LPS): n=9 - Intervention group 3 (PLV+LPS): n=9 | Perflubron | aggravation |
| S. Matsumoto et al.(56) | USA | 2004 | Organ preservation efficacy | Human clinical-grade pancreata | Total n=9: - Control group (UW group): n=6 - Intervention group (TLM group): n=3 | Perfluorochemical | effective |
| D.K. Papadimitriou et al.(64) | Greece | 2004 | Other diseases:acute intestinal ischemia | New Zealand rabbits | Total: 36 rabbits - Control group: n=12 - Intervention group (PFC-O₂ group):n=12 - Control subgroup (PFC group):n=12 Each group was further divided into 3 subgroups by ligated vessels: superior mesenteric artery, mesenteric vein, or both vessels (n=4 per subgroup) | Perfluorodecalin (F-Decalin\C₁₀F₁₈), manufactured by Fluoron GmbH, Neu-Ulm | effective |
| Markus Paxian et al.(23) | USA | 2003 | Hemorrhagic shock | male sprague-dawley rats | Total n=56: - Control groups:Sham (A): n=8; HAES (B):n=8; WB (C):n=8 ;PRBCs (D):n=8. - Intervention groups:PFC-2.7 (E):n=8;PFC-5.4 (F):n=8;PRBCs+PFC (G):n=8. | Oxygent™ | effective |
| RONALD B. HIRSCHL et al.(35) | USA | 2002 | Lung injury | adult patients | Total: 90 patients - Intervention group (PLV): n=65 - Control group (CMV): n=25 | Perflubron (LiquiVent) | Partially effective |
| Steven E. Hill et al.(58) | USA | 2002 | Normovolemic hemodilution | Adult patients undergoing elective CABG surgery with CPB | Total: n=36 - Control group (electrolyte solution): n=11;  - Intervention groups: AF0144 low-dose (1.8 g PFC/kg): n=13;  - AF0144 high-dose (2.7 g PFC/kg): n=12. | AF0144 (Perflubron emulsion, Alliance Pharmaceutical Corp, San Diego, CA, USA) | effective |
| Donat R. Spahn et al.(59) | Switzerland | 2002 | Normovolemic hemodilution | adult patients | Total n=492: - Intervention group (PFC group): n=241. - Control group (standard of care): n=251. - Subgroup (target population): n=330. | Perflubron emulsion (Oxygent™, Alliance Pharmaceutical Corp., San Diego, CA, USA) | effective |
| Robert J. Frumento et al.(19) | USA | 2002 | Safety and effectiveness verification | adult patients | Total: 9 patients - Intervention group (PFC): n=4 - Control group: n=5 | Perflubron emulsion (Oxygent™) | effective |
| Jun Sakanoue et al.(18) | Japan | 2001 | Safety and effectiveness verification | male wistar rats | Total n=21:  - Intervention group: Neo-PFC group (n=7).  - Control groups:Fluosol-DA group (n=7);BSA-Buffer group (n=7). | newly developed perfluorocarbon emulsion、Fluosol-DA | effective |
| Dirk Nolte et al.(16) | USA | 2000 | Safety and effectiveness verification | Male Syrian golden hamsters | Total: 36 - Intervention subgroups (ANH + colloid + perflubron emulsion): n=6 each - Control subgroups (ANH + colloid alone): n=6 each - Additional hypervolemic infusion groups: n=6 each | Perflubron emulsion (Oxygent, AF0144) | effective |
| SHINICHI et al.(54) | Japan | 2000 | Organ preservation efficacy | Human patients undergoing whole-pancreas transplantation | Total n=54 - Control group (UW alone group):n=44 - Intervention group (Two-layer method group):n=10 | Perfluorochemical | effective |
| Phillip T. Leese et al.(20) | USA | 2000 | Safety and effectiveness verification | healthy human volunteers | Total n=48: - Control group: n=16. - Intervention groups: P1.2 group (n=16);P1.8 group (n=16). | Oxygent™ | effective |

**Table 2. Characteristics of primary PFCs included in the review.**

| **Generation Classification** | **PFC Product** | **Particle Size** | **Core Component** | **Half-life**  **(Approx.)** | **Emulsification Method** | **Primary Scenarios** |
| --- | --- | --- | --- | --- | --- | --- |
| First Generation | Fluosol-DA | < 0.2 μm | Perfluorodecalin + Perfluorotripropylamine | ~24 h (Human) | Mechanical Emulsification (Pluronic F-68 + Egg Yolk Phospholipid) | Coronary angioplasty, intraoperative transfusion, temporary oxygen supply for acute hypoxia, suppression of lung injury inflammation, treatment of carbon monoxide poisoning |
| First Generation | Perftoran (Vidaphor) | 0.1 - 0.2 μm | Perfluorodecalin + Perfluoromethylcyclohexylpiperidine | ~24 h (Human) | High-Pressure Homogenization (Proxanol 268) | Hemorrhagic shock resuscitation, organ preservation, COVID-19-related hypoxia, hemodilution in cardiac valve replacement surgery, treatment of maxillofacial space infections, kidney transplant perfusion |
| Second Generation | Oxygent™ (AF0144) | 0.16 - 0.18 μm | Perflubron (Perfluorooctyl bromide) | ~6–9 h (Human, RES Clearance) | High-Pressure Homogenization (Egg Yolk Phospholipid) | Surgical hemodilution, CPB, allogeneic blood transfusion alternative in non-cardiac surgery, hemorrhagic shock resuscitation, gastrointestinal ischemia protection, oxygen supply support in coronary artery bypass grafting |
| Second Generation | Oxycyte | ~0.2 μm | tert-Butylperfluorocyclohexane | Unknown | High-Pressure Homogenization (Egg Yolk Phospholipid) | Acute ischemic stroke, TBI, hemodilution, protection against ischemia-reperfusion injury, treatment of arterial gas embolism |
| Second Generation | PFH (Perfluorohexyloctane) | N/A | Perfluorohexyloctane | Unknown | Mechanical Emulsification (Lecithin) | Pancreatic preservation, improvement of islet transplant survival, alleviation of ischemia-reperfusion injury during pancreatic cold storage |
| Second Generation | F6H8S5 | N/A | Perfluorohexyloctane-siloxane 5 | Unknown | Mechanical Emulsification (Egg Yolk Phospholipid) | Human pancreatic preservation, enhancement of islet oxygenation, improvement of post-transplant function, increase of islet ATP content |
| Second Generation | Perflubron | 0.16 - 0.18 μm | Perflubron (Perfluorooctyl bromide) | ~6–9 h (Human) | High-Pressure Homogenization (Egg Yolk Phospholipid) | Treatment of ARDS, liquid ventilation for lung injury, treatment of postoperative atelectasis, promotion of lung development in neonates with congenital diaphragmatic hernia |
| Third Generation | DDFPe | < 300 nm (Nano-scale) | DDFP | ~90 minutes (Human) | Nanoemulsification (Human Serum Albumin Stabilization) | Acute ischemic stroke, TBI, hemorrhagic shock resuscitation, emergency treatment of anemia, tissue preconditioning protection |
| Third Generation | A-AOCs (Albumin-derived) | < 220 nm | Albumin + Perfluorodecalin | Unknown | Ultrasonic Emulsification | Organ preservation (kidney/liver/lung), massive hemodilution, oxygen supply for ischemic tissues, perfusion protection of transplanted organs |
| Third Generation | RBC-PFC | ~170 nm | RBC Membrane + PFC | Unknown | Ultrasonic Emulsification (3 min) | Hemorrhagic shock resuscitation, maintenance of vascular stability, protection of liver and spleen tissues, oxygen supply without immune activation |
| Third Generation | PFTBA-L@GB | Nano-scale | Perfluorotributylamine + Ginkgolide B | Unknown | Self-Assembly | Ischemic stroke (synergistic intervention of thrombosis and inflammation), reduction of cerebral infarction volume, inhibition of platelet aggregation, neuroprotection |
| Third Generation | PFOB (Perfluorooctyl bromide) | Nano-scale | Perfluorooctyl bromide | 3~4 days (Human) | High-Pressure Homogenization (Egg Yolk Phospholipid) | Protection against early brain injury after subarachnoid hemorrhage, alleviation of ischemia-reperfusion injury, protection of hepatocyte mitochondrial function |
| Third Generation | Perfluorodecalin (PFD) | N/A | Perfluorodecalin | 7–8 days (Tissue) | Mechanical Emulsification (Lecithin) | Prolongation of intestinal tissue viability in acute intestinal ischemia, clearance of airway thrombi in pulmonary hemorrhage, assisted ventilation for neonatal respiratory distress syndrome |
